# The Interspecific Fungal Hybrid *Verticillium longisporum* Displays Subgenome-Specific Gene Expression

**DOI:** 10.1128/mBio.01496-21

**Published:** 2021-07-20

**Authors:** Jasper R. L. Depotter, Fabian van Beveren, Luis Rodriguez-Moreno, H. Martin Kramer, Edgar A. Chavarro Carrero, Gabriel L. Fiorin, Grardy C. M. van den Berg, Thomas A. Wood, Bart P. H. J. Thomma, Michael F. Seidl

**Affiliations:** a Laboratory of Phytopathology, Wageningen University and Research, Wageningen, The Netherlands; b Department of Crops and Agronomy, National Institute of Agricultural Botany, Cambridge, United Kingdom; c University of Cologne, Institute for Plant Sciences, Cluster of Excellence on Plant Sciences (CEPLAS), Cologne, Germany; d Theoretical Biology and Bioinformatics, Utrecht University, Utrecht, The Netherlands; e Departamento de Biología Celular, Genética y Fisiología, Universidad de Málaga, Málaga, Spain; Duke University

**Keywords:** allopolyploidization, *Verticillium* stem striping, genome rearrangements, gene conversion, haploidization, mosaic genome, chromatin conformation capture (Hi-C)

## Abstract

Hybridization is an important evolutionary mechanism that can enable organisms to adapt to environmental challenges. It has previously been shown that the fungal allodiploid species Verticillium longisporum, the causal agent of verticillium stem striping in rapeseed, originated from at least three independent hybridization events between two haploid *Verticillium* species. To reveal the impact of genome duplication as a consequence of hybridization, we studied the genome and transcriptome dynamics upon two independent V. longisporum hybridization events, represented by the hybrid lineages “A1/D1” and “A1/D3.” We show that *V. longisporum* genomes are characterized by extensive chromosomal rearrangements, including between parental chromosomal sets. *V. longisporum* hybrids display signs of evolutionary dynamics that are typically associated with the aftermath of allodiploidization, such as haploidization and more relaxed gene evolution. The expression patterns of the two subgenomes within the two hybrid lineages are more similar than those of the shared A1 parent between the two lineages, showing that the expression patterns of the parental genomes homogenized within a lineage. However, as genes that display differential parental expression *in planta* do not typically display the same pattern *in vitro*, we conclude that subgenome-specific responses occur in both lineages. Overall, our study uncovers genomic and transcriptomic plasticity during the evolution of the filamentous fungal hybrid *V. longisporum* and illustrates its adaptive potential.

## INTRODUCTION

Upon hybridization, two distinct genotypes are merged into a single organism. This surge in genomic variation can increase the adaptive potential of hybrid organisms, which may explain why stable hybrids are generally more fit than their parents in particular environments (1). However, hybrids may also encounter incompatibilities between parental genomes as they lack the recently shared evolutionary history (2). Hybridization can lead to the emergence of new species that are reproductively isolated from their parents, known as hybrid speciation (3, 4). Although the incidence of hybridization may be rare due to such incompatibilities, many organisms encountered hybridization at a particular point in their evolution (5). Hybridization has also impacted the evolution of humans, as our genomes still contain traces of Neanderthal introgression (6). Hybridization can occur between gametes after conventional meiosis, leading to so-called homoploid hybrids. Alternatively, when complete sets of parental chromosomes combine, hybridization is accompanied by genome duplication during so-called allopolyploidization.

Hybridization has impacted the evolution of a wide diversity of fungi (7–9). For instance, the yeast Saccharomyces paradoxus, a close relative of the baker’s yeast Saccharomyces cerevisiae, has naturally hybridized in North American forests (10), whereas S. cerevisiae itself was also shown to have undergone an ancient interspecies hybridization event (11). Similarly, various *Candida* species that are opportunistic human pathogens display genomic traces of hybridization events (12–15). Hybridization also contributed to the evolution of various plant-pathogenic fungi (7). Plant pathogens generally coevolve with their hosts to evade host immunity, while hosts attempt to intercept pathogen ingress (16). In this process, plant pathogens secrete effector proteins that contribute to host immunity evasion and interfere with host metabolic processes (17) or affect other processes to contribute to host colonization (18), such as the manipulation of host microbiomes (19, 20). Due to the increased adaptation potential, hybridization has been proposed as a potent driver in pathogen evolution as it can impact host interactions through increased virulence and host range alterations (8). For instance, the Ug99 strain of the wheat stem rust pathogen Puccinia graminis f. sp. *tritici* arose from a hybridization event and caused devastating epidemics in Africa and the Middle East (21, 22). Recent hybridization between wheat powdery mildew, Blumeria graminis f. sp. *tritici*, and rye powdery mildew, *B. graminis* f. sp. *secalis*, gave rise to the novel mildew species B. graminis f. sp. *triticale*, which, in contrast to its parents, is able to cause disease on triticale (23).

Upon hybridization, genomes typically experience a so-called “genome shock,” inciting major genomic reorganizations that can manifest as genome rearrangements, extensive gene loss, transposon activation, and alterations in gene expression (24, 25). Conceivably, these early-stage alterations are primordial for hybrid survival, as divergent evolution is principally associated with incompatibilities between the parental genomes (26). Additionally, these initial reorganizations and further alterations in the aftermath of hybridization provide a source for environmental adaptation. Frequently, hybrid genomes lose their heterozygosity over time (27). Hybrids that are still sexually compatible with one of their parents can lose heterozygosity through backcrossing. Alternatively, heterozygosity can be a result of the direct loss of a homolog of one of the two parents (i.e., a homeolog) through deletion or gene conversion whereby one of the copies replaces its homeologous counterpart. Gene conversion and the homogenization of complete chromosomes played a pivotal role in the evolution of the osmotolerant yeast species Pichia sorbitophila (28). Two of its seven chromosome pairs consist of partly heterozygous and partly homozygous sections, whereas two chromosome pairs are completely homozygous. Gene conversion may eventually result in chromosomes consisting of sections of both parental origins, so-called “mosaic genomes” (29). However, mosaic genomes can also arise through recombination between chromosomes of the different parents, such as in the hybrid yeast Zygosaccharomyces parabailii (30). Hybridization associated with polyploidy, allopolyploids, can have additional adaptive potential through the presence of an additional copy for most genes, which gives leeway to functional diversification (31, 32). Hybridization typically also entails alterations of gene expression patterns that are nonadditive from the parental expression patterns (33, 34). Nevertheless, expression patterns are generally conserved upon hybridization, as the majority of allopolyploid genes are expressed in a fashion similar to that of their parental orthologs (35). For instance, more than half of the genes in an allopolyploid strain of the fungal grass endophyte *Epichloë* retained their parental gene expression pattern (36). Similar conservation has also been observed for *Blumeria graminis* f. sp. *triticale* as over half of the 5% most highly expressed genes are shared with both of its hybridization parents (37). In conclusion, the genomic and transcriptomic alterations accompanied by hybridization make hybrids have a high potential for environmental adaptation (8).

Within the *Verticillium* genus that comprises nine haploid species, hybridization resulted in the emergence of the species Verticillium longisporum (38–41). V. longisporum is subdivided into three lineages, each representing a separate hybridization event (39, 41). *Verticillium* species A1 is a parent of each of the three hybrids and hybridized with *Verticillium* species D1, D2, and D3, resulting in the *V. longisporum* lineages A1/D1, A1/D2, and A1/D3, respectively. Whereas species D2 and D3 have been classified as “likely Verticillium dahliae,” species D1 has been classified as an enigmatic species that is closely related to V. dahliae (39). Species A1 is also an enigmatic species that diverged from V. dahliae earlier than the D1 species (39). Similar to the haploid *Verticillium* species, *V. longisporum* is thought to mainly undergo asexual reproduction, as a sexual cycle has never been described, and populations are not outcrossing (40, 41). Interestingly, *V. longisporum* mainly infects plant hosts of the Brassicaceae family, whereas other *Verticillium* species do not cause disease on brassicaceous hosts (42). Moreover, while V. dahliae is characterized by an extremely broad host range that comprises hundreds of (non-Brassicaceae) plant species, *V. longisporum* has only a limited host range and hardly infects non-Brassicaceae species (42). After hybridization, *V. longisporum* conceivably encountered extensive genetic and transcriptomic alterations that facilitated its viability as a hybrid and the shift toward brassicaceous hosts. In this study, we investigated the impact of allodiploidization on the evolution of *V. longisporum* by investigating genome, gene, and transcriptomic plasticity within and between two of the hybridization events.

## RESULTS

### *Verticillium longisporum* displays a mosaic genome structure.

**FIG 1:**
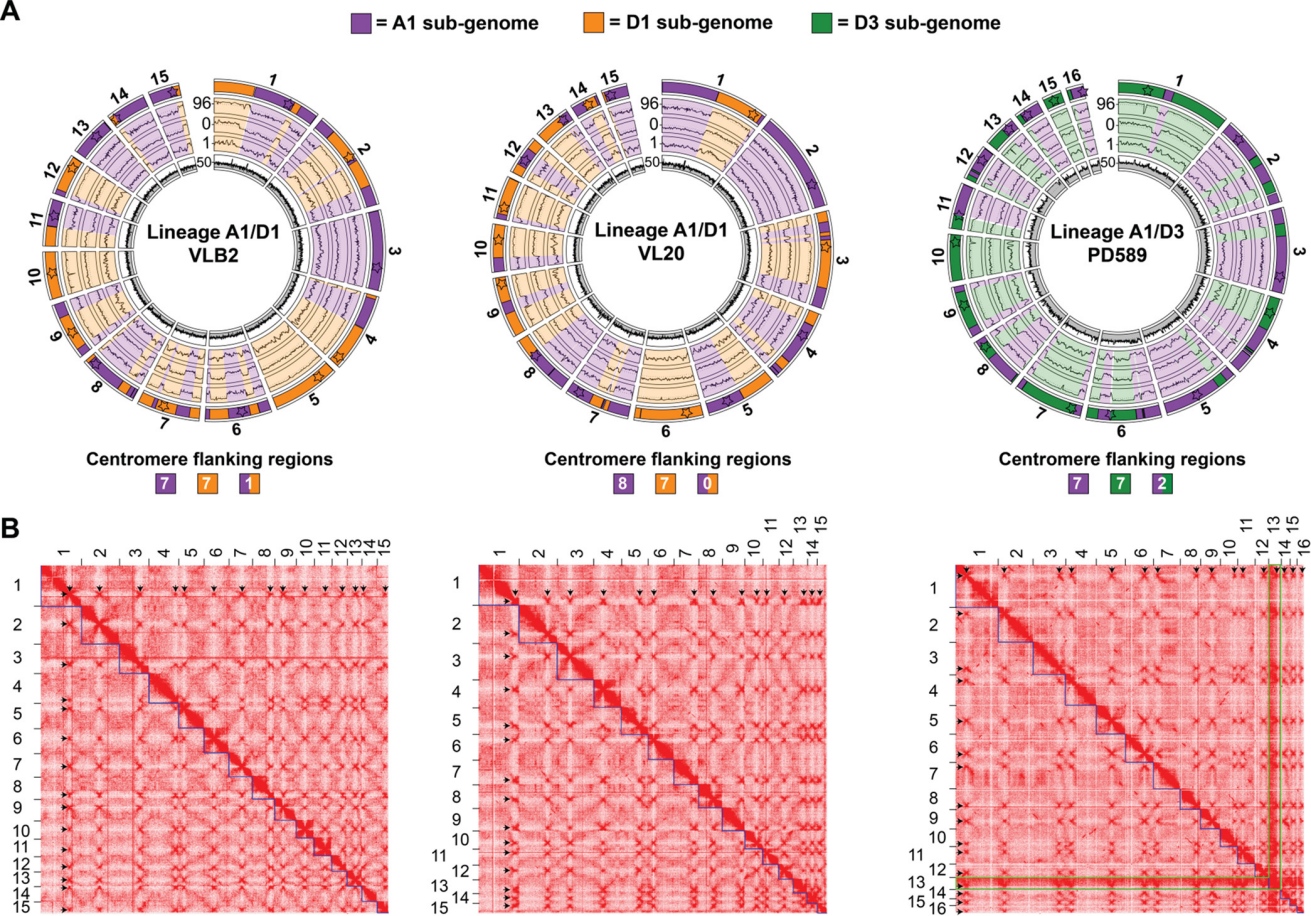
*Verticillium longisporum* displays a mosaic genome structure. (A) *V. longisporum* chromosomes of strains VLB2, VL20, and PD589. The different lanes in the circular plots represent (i) regions assigned to species A1, species D1, and species D3, with the stars representing the locations of the centromeres and their color representing the parental origin based on their flanking regions; (ii) sequence similarity of *V. longisporum* alignments to V. dahliae (percent identity); (iii) differences in sequence identity in percentage points (pp) between exonic regions of *V. longisporum* double-copy genes, where only gene pairs with an ortholog in V. dahliae are depicted and alleles with a higher identity to V. dahliae are depicted as a positive pp difference, whereas the corresponding homolog is depicted as a negative pp difference; (iv) the relative difference in GC content (dGC) between genes in double copy; and (v) read depth with nonoverlapping windows of 10 kb. Data points of lanes 3 to 5 represent the average values for a window of 11 genes, which proceed with a step of 1 gene. (B) Hi-C contact frequency matrices for the three *V. longisporum* strains. Red indicates the contact intensity between genome regions, and the blue squares represent the pseudochromosomes. Centromeres display strong interchromosomal contacts and are visible as red dots outside the pseudochromosomes and indicated with black arrows. Pseudochromosome 13 of strain PD589 generally displays stronger interactions than the other pseudochromosomes and is outlined in green.

The genomes of three *V. longisporum* strains from two different hybridization events were analyzed to investigate the impact of hybridization on genome structure. Previously, *V. longisporum* strains VLB2 and VL20, both belonging to the A1/D1 hybridization event, were sequenced with the PacBio RSII platform and assembled *de novo* (40). We now additionally sequenced *V. longisporum* strain PD589, which originates from the A1/D3 hybridization event (39), using Oxford Nanopore Technologies (ONT) sequencing technology and the BGISeq platform to obtain long reads and paired-end short reads, respectively. All *V. longisporum* genome assemblies were improved using chromatin conformation capture (Hi-C) sequencing that detects DNA interactions (43) (see Fig. S1 in the supplemental material). Moreover, centromeres can be located with Hi-C sequencing as they display strong interactions with centromeres in other chromosomes (44) (Fig. S1). We obtained genome assemblies of 72.7, 72.2, and 72.0 Mb consisting of 15, 15, and 16 pseudochromosomes for VLB2, VL20, and PD589, respectively (Fig. 1A and Table 1). Every pseudochromosome contained a centromere, suggesting that the A1/D1 isolates have 15 chromosomes and that the A1/D3 isolate PD589 contains 16 chromosomes (Fig. 1A). However, chromosome 13 of strain PD589 displayed remarkably stronger DNA interactions than the other chromosomes (Fig. 1B, green outline), as the median read coverage of chromosome 13 is 110×, whereas the read coverage is 58× to 70× for all other chromosomes (Fig. S2). This finding suggests that chromosome 13 recently (partly) duplicated since the high sequence identity of the duplicated regions resulted in a collapsed assembly. Consequently, strain PD589 may therefore actually have 17 chromosomes in total.

**TABLE 1:** Comparison of *Verticillium longisporum* and Verticillium dahliae genome assemblies

Parameter	Value for assembly			
	*V. longisporum* VLB2^*a*^	*V. longisporum* VL20^*a*^	*V. longisporum* PD589	V. dahliae JR2^*b*^
Genome size (Mb)	72.7	72.2	72.0	36.2
Assigned to A1 subgenome	36.2	36.5	36.0	
Assigned to D subgenome	35.9	35.1	34.7	
Undetermined	0.6	0.6	1.3	
No. of chromosomes	15	15	16/17^*d*^	8
No. of predicted genes	18,679	18,592	18,251	9,636
Assigned to A1 subgenome	9,342	9,343	8,961	
Assigned to D subgenome	9,298	9,188	9,229	
Undetermined	39	61	61	
No. of predicted genes encoding secreted proteins	2,084	2,049	1,960	1,071
Assigned to A1 subgenome	1,052	1,041	952	
Assigned to D subgenome	1,025	1,004	1,000	
Undetermined	7	4	8	
Repeat content (%)	14.55	14.54	12.78	11.69
BUSCO completeness (%)^*c*^	99.1	99.3	97.9	98.6

a Previously published assemblies were reassembled using Hi-C sequencing (40).

b See reference 47.

c Based on Ascomycota benchmarking universal single-copy orthologs (BUSCOs).

d The total chromosome number is uncertain as PD589 contains one (partially) duplicated chromosome.

10.1128/mBio.01496-21.1 FIG S1 *Verticillium longisporum* strain PD589 displays a mosaic genome structure based on Hi-C data. The proximity of the chromosome 2 sequences to other sequences in the genome is indicated by the intensity of the red color. In the plot of chromosome 2 with itself, a continuous red band can be observed from the upper left to the bottom right corner, which is absent from all other chromosome comparisons. This band indicates that chromosome 2 sequences reside in proximity to the sequences that neighbor them in the assembly. Chromosome 2 consists of genome regions with A1 (purple) and D3 (green) origins. Consequently, the Hi-C data demonstrate that genomic regions of different parental origins reside next to each other on a single chromosome. Apart from the continuous band in the self-alignment, chromosome 2 also displays proximity to the other chromosomes in one particular region, namely, centromeres (indicated by arrows). Centromeres are known to display interchromosomal interactions and reside in proximity to each other in the nucleus. Download FIG S1, PDF file, 0.9 MB. Copyright © 2021 Depotter et al. 2021 Depotter et al. https://creativecommons.org/licenses/by/4.0/ This content is distributed under the terms of the Creative Commons Attribution 4.0 International license.

10.1128/mBio.01496-21.2 FIG S2 Read coverage for the *Verticillium longisporum* strain PD589 genome assembly. The red lines indicate the average coverage of Oxford Nanopore sequenced reads for 20-kb windows. The blue line indicates the median coverage for every individual chromosome. Download FIG S2, PDF file, 0.3 MB. Copyright © 2021 Depotter et al. 2021 Depotter et al. https://creativecommons.org/licenses/by/4.0/ This content is distributed under the terms of the Creative Commons Attribution 4.0 International license.

Being able to determine the parental origin of individual genomic regions is elementary to investigating genome evolution in the aftermath of hybridization. As the D parents of *V. longisporum* hybridizations (D1 and D3) are phylogenetically more closely related to V. dahliae than parent A1 (39), *V. longisporum* genome alignments to V. dahliae display a bimodal distribution with minima at ∼96.0% identity (Fig. S3A). Similarly, the sequence identity between coding regions of *V. longisporum* and V. dahliae orthologs displayed a bimodal distribution, with minima that are less pronounced than those of the genome alignments (Fig. S3B). To separate the two subgenomes, we used genome alignments and coding region sequence identities. Genome regions were assigned to parent A1 if their average sequence identity to V. dahliae was lower than this minimum to parent A1 and/or their coding regions displayed 93 to 98.5% sequence identity with their V. dahliae ortholog (Fig. S3). In contrast, regions with alignments and/or coding regions with higher sequence identities were assigned to the D parent (Fig. S3). In this manner, 36.0 to 36.5 Mb were assigned to the A1 parents, and 34.7 to 35.9 Mb were assigned to the D parents (Fig. 1A and Table 1). Thus, the subgenome sizes are quite similar for each of the isolates and correspond to the expected genome sizes of haploid *Verticillium* species (44, 45). As the three *V. longisporum* strains have the A1 parent in common, we used nucleotide substitutions in the A1 coding regions to roughly estimate the minimum divergence time of the *V. longisporum* strains since their last common ancestor. Estimates of nucleotide mutation rates are as yet not available for *Verticillium*, so we used the estimated asexual mutation rate for the coding region of Neurospora crassa (9.05 × 10^−9^ per site per day or 6.03 × 10^−10^ per site per cell division) (46). Between strains VLB2 and VL20 from the same hybridization event, 0.073% of the nucleotides of their A1 homologs displayed substitutions, which corresponds to 1,204,419 cell divisions or 220 years of continuous growth on culture medium. In contrast, between hybridization events, PD589 homologs displayed 0.24 and 0.23% nucleotide substitutions with VLB2 and VL20 homologs, respectively, which correspond to 3,899,045 and 3,845,760 cell divisions or 712 and 702 years of continuous growth on culture medium, respectively. As *Verticillium* does not grow continuously under optimal conditions in nature and produces microsclerotia to overwinter in the absence of the host, which can remain dormant and viable for more than 10 years when hosts are not available in the next growing season (41), these divergence time estimates are certainly severe underestimations of the actual divergence times and should be considered minimum divergence times.

10.1128/mBio.01496-21.3 FIG S3 Evidence used for the A1 and D subgenome division of the *Verticillium longisporum* strains. (A) Density distribution of sequence identities of *V. longisporum* alignments to Verticillium dahliae. *V. longisporum* strains were aligned to V. dahliae JR2 with NUCmer. The average sequence identity of these alignments was used to determine smoothed density estimates. (B) Density distribution of sequence identities of *V. longisporum* gene coding regions to their V. dahliae orthologs. The sections indicated in red were assigned to the A1 subgenome, whereas the blue ones were assigned to the D subgenomes. Download FIG S3, PDF file, 0.5 MB. Copyright © 2021 Depotter et al. 2021 Depotter et al. https://creativecommons.org/licenses/by/4.0/ This content is distributed under the terms of the Creative Commons Attribution 4.0 International license.

The majority of the *V. longisporum* chromosomes are composed of DNA regions that originate from different parents, and only two chromosomes have a single parental origin in each of the strains (Fig. 1; Table S1). Using different genome assembly approaches, the genomic regions of different parental origins were consistently assembled together, excluding the possibility that this phenomenon is caused by assembly artifacts (Fig. S4). Thus, *V. longisporum* chromosomes generally are mosaics of DNA regions of different parental origins. As genomic rearrangements may also occur over centromeres (44), we assessed if such rearrangements could be identified. One centromere of VLB2 and two of PD589 are flanked by regions of differential parental origins, demonstrating that rearrangements between the parental genomes occurred over these centromeres (Fig. 1A). In contrast, strain VL20 did not have centromeres flanked by regions of different parental origins (Fig. 1A).

10.1128/mBio.01496-21.4 FIG S4 The mosaic appearance of the *Verticillium longisporum* assemblies is not caused by assembly artifacts. VLB2 and VL20 *V. longisporum* chromosomes are depicted, which were assembled using Hi-C data. In the first lane of the circular plot, the parental origin of genomic regions is indicated. In the second lane, the previously published *V. longisporum* assemblies were aligned to chromosomes (J. R. L. Depotter, M. F. Seidl, G. C. M. van den Berg, B. P. H. J. Thomma, and T. A. Wood, Environ Microbiol 19:3997–4009, 2017, https://doi.org/10.1111/1462-2920.13801). Points of discontinuity in the alignments that correspond to a genome location where A1 and D1 sequences reside next to each other are indicated with a green dot (3 for VLB2 and 2 for VL20). In the third lane, subgenome *V. longisporum* assemblies were aligned to the chromosomes. To this end, PacBio reads from different parental origins were separated from each other and separately assembled using Canu v2 (S. Koren, B. P. Walenz, K. Berlin, J. R. Miller, et al., Genome Res 27:722–736, 2017, https://doi.org/10.1101/gr.215087.116). Genomic regions that are connected in the subgenome assembly and not in the Hi-C assembly are indicated with ribbons in the center of the circular plot. The point where the genome regions are connected to each other is indicated in the ribbons by the color blue. The locations where the mismatch in the Hi-C and subgenome assembly corresponds to a genome location where A1 and D1 sequences reside next to each other are indicated with a red dot (1 for VLB2 and 2 for VL20). PacBio reads were mapped to the genome locations where green and red dots were identified to see putative discontinuities in the read mapping that would indicate putative assembly errors. No such discontinuities were observed, as for every location, multiple reads bridged the A1 and D1 genomic regions. Hence, all observed genomic rearrangements are supported by overlapping PacBio reads and thus are not the consequence of assembly artifacts. Download FIG S4, PDF file, 1.4 MB. Copyright © 2021 Depotter et al. 2021 Depotter et al. https://creativecommons.org/licenses/by/4.0/ This content is distributed under the terms of the Creative Commons Attribution 4.0 International license.

10.1128/mBio.01496-21.8 TABLE S1 Fractions of individual *Verticillium longisporum* chromosomes that belong to the A1 and D parents. Download Table S1, DOC file, 0.05 MB. Copyright © 2021 Depotter et al. 2021 Depotter et al. https://creativecommons.org/licenses/by/4.0/ This content is distributed under the terms of the Creative Commons Attribution 4.0 International license.

### The mitochondrial genome is inherited from the A1 parent in all lineages.

To determine the phylogenetic position of the parental subgenomes of *V. longisporum*, we used the *V. longisporum* subgenome sequences and previously published genome sequences of the haploid *Verticillium* species (45, 47) to construct a phylogenetic tree based on 1,520 ascomycete benchmarking universal single-copy orthologs (BUSCOs) that were present in a single copy in all analyzed *Verticillium* lineages. In accordance with previous phylogenetic studies (39, 40), the A1 parents diverged earlier from V. dahliae than the D1 and D3 parents (Fig. S5). Furthermore, the D1 parent diverged earlier from V. dahliae than the D3 parent. We also constructed a phylogenetic tree based on mitochondrial DNA to determine the parental origin of the mitochondria. The *V. longisporum* mitochondrial genomes were assembled in a single contig with overlapping ends, indicating their circular nature. The mitochondrial genomes of the three *V. longisporum* strains were all 26.2 kb in size and were more than 99.9% identical in sequence. The phylogenetic position of the *V. longisporum* mitochondrial genomes clusters with the mitochondrial genomes of V. alfalfae and V. nonalfalfae (Fig. S5). As the mitochondrial genome sequences are almost identical for three strains that are derived from the two hybridization events, the common A1 parent is the likely donor of the mitochondria.

10.1128/mBio.01496-21.5 FIG S5 Phylogenetic relationships between *Verticillium longisporum* hybridization parents and haploid *Verticillium* species. A tree of the nuclear genomes was constructed based on 1,520 ascomycete benchmarking universal single-copy orthologs (BUSCOs) that are present in a single copy in all analyzed *Verticillium* lineages. A tree of the mitochondrial genomes was constructed based on the complete sequence of the mitochondrial genome. The robustness of the inferred phylogeny was assessed by 100 bootstrap approximations. Download FIG S5, PDF file, 0.2 MB. Copyright © 2021 Depotter et al. 2021 Depotter et al. https://creativecommons.org/licenses/by/4.0/ This content is distributed under the terms of the Creative Commons Attribution 4.0 International license.

### Genomic rearrangements are responsible for the mosaic nuclear genome.

**FIG 2:**
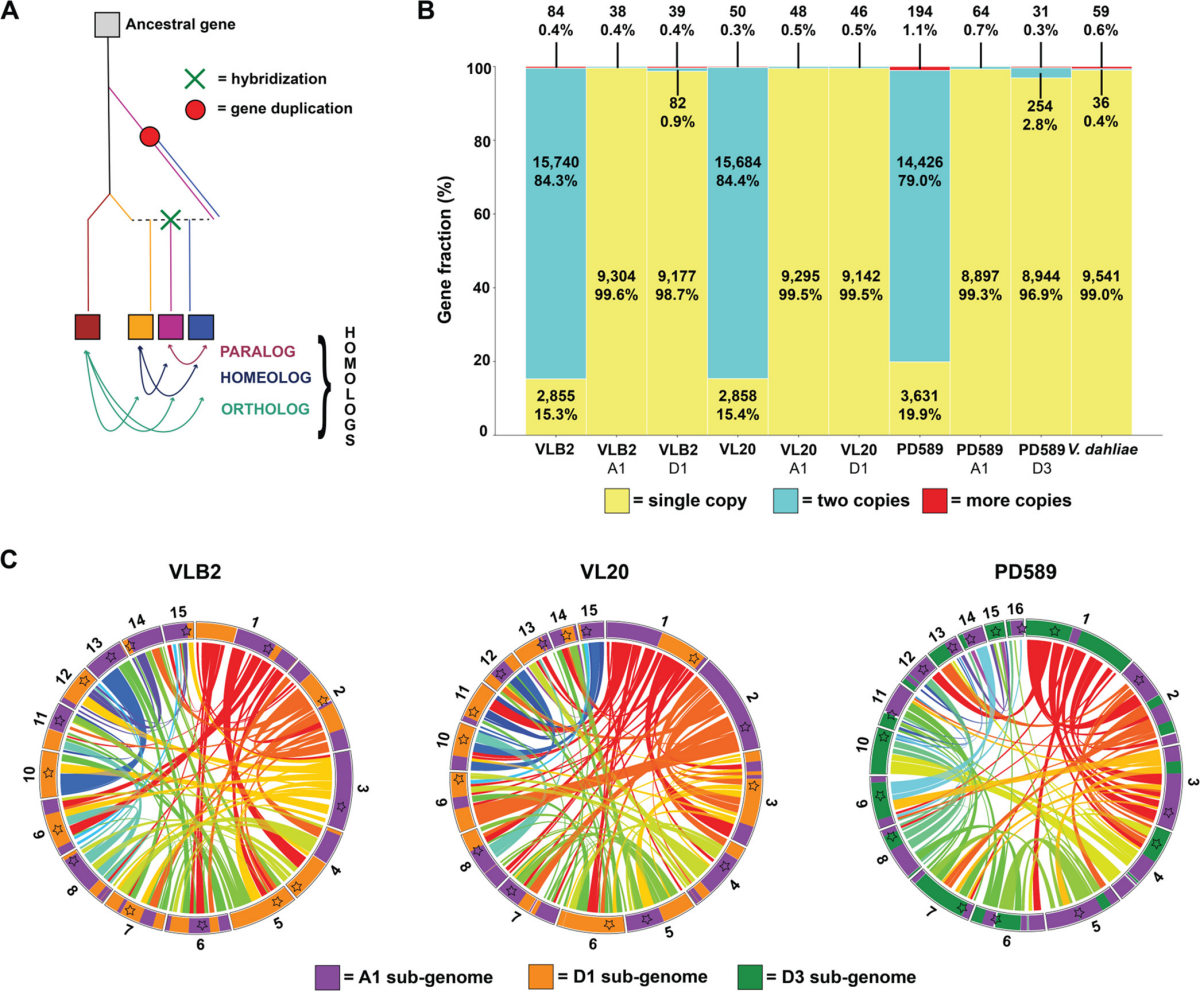
*Verticillium longisporum* genes with two copies are almost exclusively homeologs. (A) Schematic overview of different evolutionary origins of homologous genes in hybrids. Paralogs are homologous genes that originate from gene duplication, while orthologous genes originate by speciation. Homeologs are homologous genes originating from a hybridization event. (B) The gene fractions occurring in single, two, and more than two copies in *V. longisporum* strains VLB2, VL20, and PD589, with V. dahliae (strain JR2) as a comparison. “A1,” “D1,” and “D3” represent species A1, D1, and D3 subgenomes, respectively. (C) Homeologous regions within the *V. longisporum* genomes. Ribbons indicate homeologous regions within the strains, and contig colors indicate the parental origins, similar to Fig. 1.

**FIG 3:**
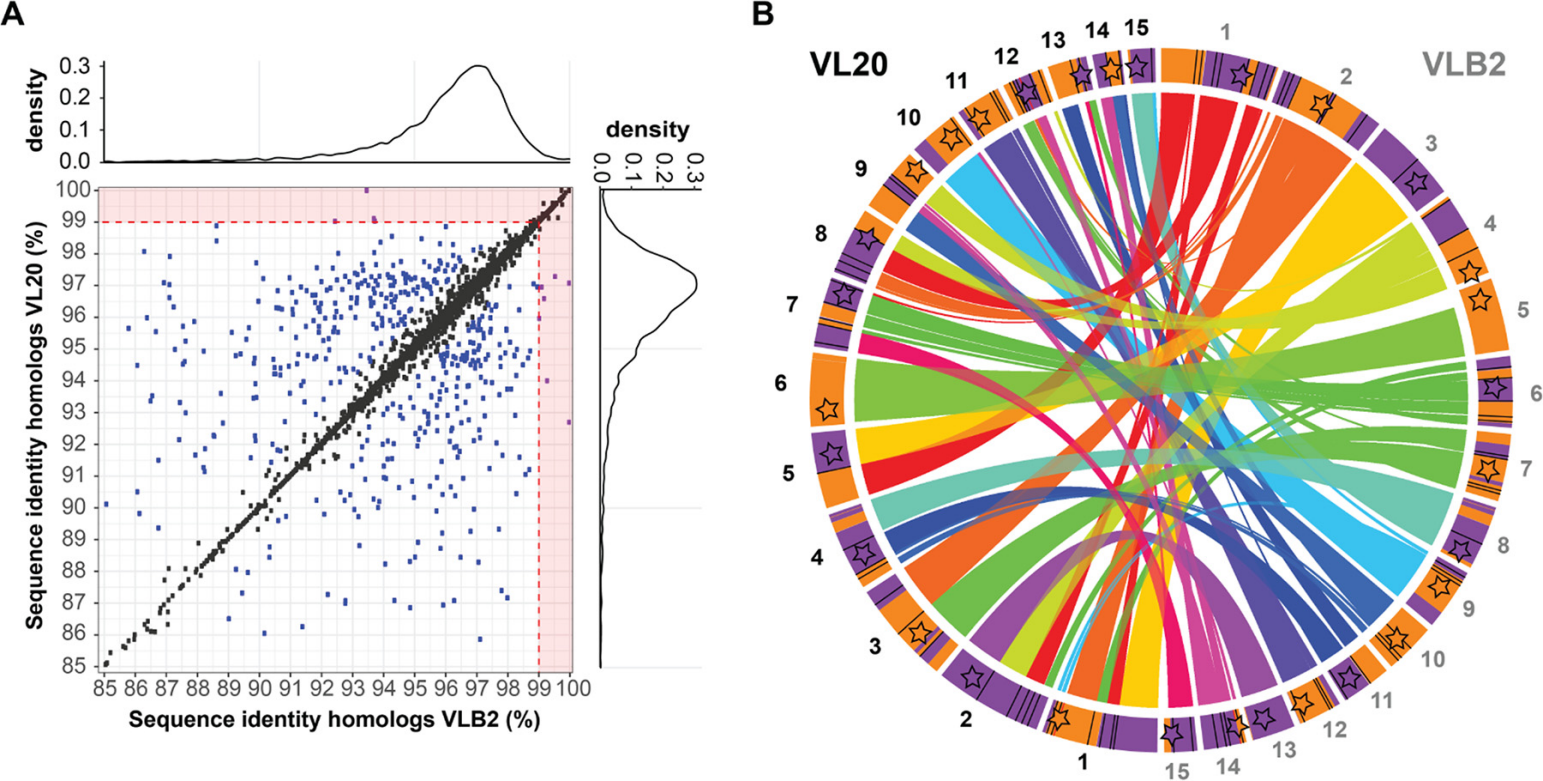
The mosaic genome structure of *Verticillium longisporum* originates from genomic rearrangements. (A) Contribution of gene conversion to *V. longisporum* genome evolution. Sequence identities between genes in copy, present in *V. longisporum* VLB2 and VL20, are depicted. Homologous gene pairs within a strain that encountered gene conversion are expected to have higher similarity within a strain than with the corresponding gene pair in the other strain. Gene pairs with a divergence of more than 1% in one *V. longisporum* strain and less than 1% in the other strain were considered conserved in the latter strain (purple dots in the red zones). In other cases, pairs that differ by less than 1% are depicted as a black dot, whereas a difference greater than 1% is depicted as a blue dot. (B) Contribution of genomic rearrangements to *V. longisporum* genome evolution. The *V. longisporum* chromosomes of strains VLB2 (right) and VL20 (left) are depicted. Ribbons indicate syntenic genome regions between the two strains, and contig colors indicate the parental origin, similar to Fig. 1 (purple, A1; orange, D1). Black lines on the chromosomes indicate synteny breaks that are not associated with centromeres, whereas red ones are associated with centromeres.

Typically, a mosaic structure of a hybrid nuclear genome can originate from gene conversion or chromosomal rearrangements between DNA strands of different parental origins (27). To analyze the extent of gene conversion, protein-coding genes were predicted for the *V. longisporum* strains using BRAKER with RNA sequencing (RNA-Seq) data from fungal cultures grown *in vitro* (48). The number of predicted genes ranged from 18,251 to 18,679 for the different *V. longisporum* strains, which is 89 to 94% higher than the gene number of V. dahliae strain JR2 predicted using the same methodology (9,636 genes) (Table 1). In total, 8,961 to 9,343 genes were assigned to the subgenome of parent A1, whereas the number of genes in the D3 subgenomes ranged from 9,188 to 9,298 (Table 1). Thus, the gene numbers are similar for the different *V. longisporum* subgenomes and comparable to the gene number of V. dahliae. Over 79% of the *V. longisporum* genes have one homolog; i.e., they occur in two copies, which can originate from gene duplication (paralogy) or the hybridization event (homeology) (Fig. 2A and B). Within each of the *V. longisporum* subgenomes, most genes (96.9 to 99.6%) have no additional homolog and occur in a single copy (Fig. 2B), indicating that most homologous gene pairs in each *V. longisporum* genome are homeologous in nature and that gene conversion played only a minor role after hybridization. Accordingly, over 80% of the D subgenomes have one homeologous genome region (Fig. 2C). To find traces of gene conversion during their evolution, the sequence identities of 6,213 genes that have two homologous copies in the two A1/D1 strains were compared, as these two strains belong to distinct populations (40). Only seven genes were found to be highly similar (<1% nucleotide sequence diversity) in VLB2, whereas the corresponding gene pair in VL20 was more diverse (>1%) (Fig. 3A). Similarly, in *V. longisporum* strain VL20, four highly similar copies were found that are more divergent in VLB2, thereby confirming that gene conversion has hitherto played only a marginal role in the evolutionary aftermath of *V. longisporum* hybridization.

Considering that gene conversion played only a minor role during genome evolution (Fig. 3), the mosaic genome structure of *V. longisporum* likely originated from rearrangements between homeologous chromosomes. To identify chromosomal rearrangements after the hybridization event that led to the A1/D1 lineage, the genome of *V. longisporum* strain VLB2 was aligned to that of strain VL20, revealing 46 syntenic breaks (Fig. 3B). Rearrangement occurred in the majority of the chromosomes as only 2 and 1 chromosomes did not have syntenic breaks in VLB2 and VL20, respectively (Fig. 3B). As genomic rearrangements are often associated with repeat-rich genome regions, such as in V. dahliae (47, 49, 50), the synteny breakpoints were tested for their association with repetitive regions. Since the median repeat fraction in a 20-kb window around the repeats is 10.8%, which is significantly higher than the median repeat fraction based on random sampling (average = 3.1%; σ = 0.78%) (Fig. S6), it can be concluded that the chromosomal rearrangements are also similarly associated with repeats in *V. longisporum*. Furthermore, of the 46 breakpoints between VLB2 and VL20, 1 is associated with a VLB2 centromere, and 2 are associated with a VL20 centromere (Fig. 3B, red lines). In conclusion, chromosomal rearrangement rather than gene conversion is the main mechanism explaining the mosaic structure of the *V. longisporum* genome.

10.1128/mBio.01496-21.6 FIG S6 Association of synteny breaks with repetitive elements. The black curve represents the normalized density of the median repeat content of 46 randomly chosen 20-kb windows in *Verticillium longisporum* VL20, which has been permutated 10,000 times. The red line indicates the median repeat content of 20-kb windows around 46 synteny breaks between VLB2 and VL20 (10.8%). The red region indicates the 5% highest data points. Download FIG S6, PDF file, 0.1 MB. Copyright © 2021 Depotter et al. 2021 Depotter et al. https://creativecommons.org/licenses/by/4.0/ This content is distributed under the terms of the Creative Commons Attribution 4.0 International license.

### *V. longisporum* loses heterozygosity through deletions.

To study putative gene losses in the aftermath of hybridization, we determined genes that have no homeolog or paralog and can thus be considered to occur in a single copy. For the A1/D1 isolates, 15.3 to 15.4% of the genes occur in a single copy, whereas this fraction is 19.9% for A1/D3 isolate PD589 (Fig. 2B). We checked if proteins encoded by single-copy genes are enriched for particular Gene Ontology (GO) terms or Clusters of Orthologous Groups (COGs) or encode a protein with a signal peptide, which suggests that these proteins are secreted. No GO terms or COGs were enriched for the single-copy genes in any of the *V. longisporum* strains (*P* value of <0.05 by Fisher’s exact test with Benjamini-Hochberg correction). In total, 7.8 to 10.2% of the single-copy genes encode a protein with a signal peptide, which is significantly lower than the 11.9 to 12.3% for genes with a homologous copy in the same genome (*P* value of <0.05 by Fisher’s exact test). Of the A1/D1 single-copy genes, 52% reside in the A1 subgenome, and 47% reside in the D1 subgenome. Similarly, for PD589, 49% and 50% reside in the A1 and D3 subgenomes, respectively. Thus, single-copy genes are equally distributed across the two subgenomes in *V. longisporum*. Single-copy genes can originate from either gene loss or parent-specific contributions to the hybrid. Since VLB2 and VL20 originate from the same hybridization event (40), we can quantify how many single-copy genes originate from gene loss during the divergence of VLB2 and VL20. In total, 14.7 to 14.8% of the singly-copy genes have at least one copy in each subgenome of the other A1/D1 strain, suggesting that gene deletion is an ongoing process in *V. longisporum* evolution. Of the single-copy genes that lost their homeolog after the hybridization event, 48% resided in the species A1 subgenome, whereas 51 to 52% resided in the D1 subgenome, suggesting that gene losses occurred to similar extents in each of the subgenomes. Furthermore, 24% (14 clusters) and 15% (13 clusters) of genes in VLB2 and VL20, respectively, that are lost after the divergence of these two strains are clustered in the other strain, indicating that large deletions occur. The gene clusters lost in VL20 do not localize at any of the 46 synteny breakpoints between VLB2 and VL20 (Fig. 3B). In contrast, 4 of the 14 clusters that are lost in VLB2 are associated with such breakpoints. Thus, genomic recombination may lead to the loss of gene clusters but does not explain the majority of the gene losses.

### Acceleration of gene evolution upon hybridization.

**FIG 4:**
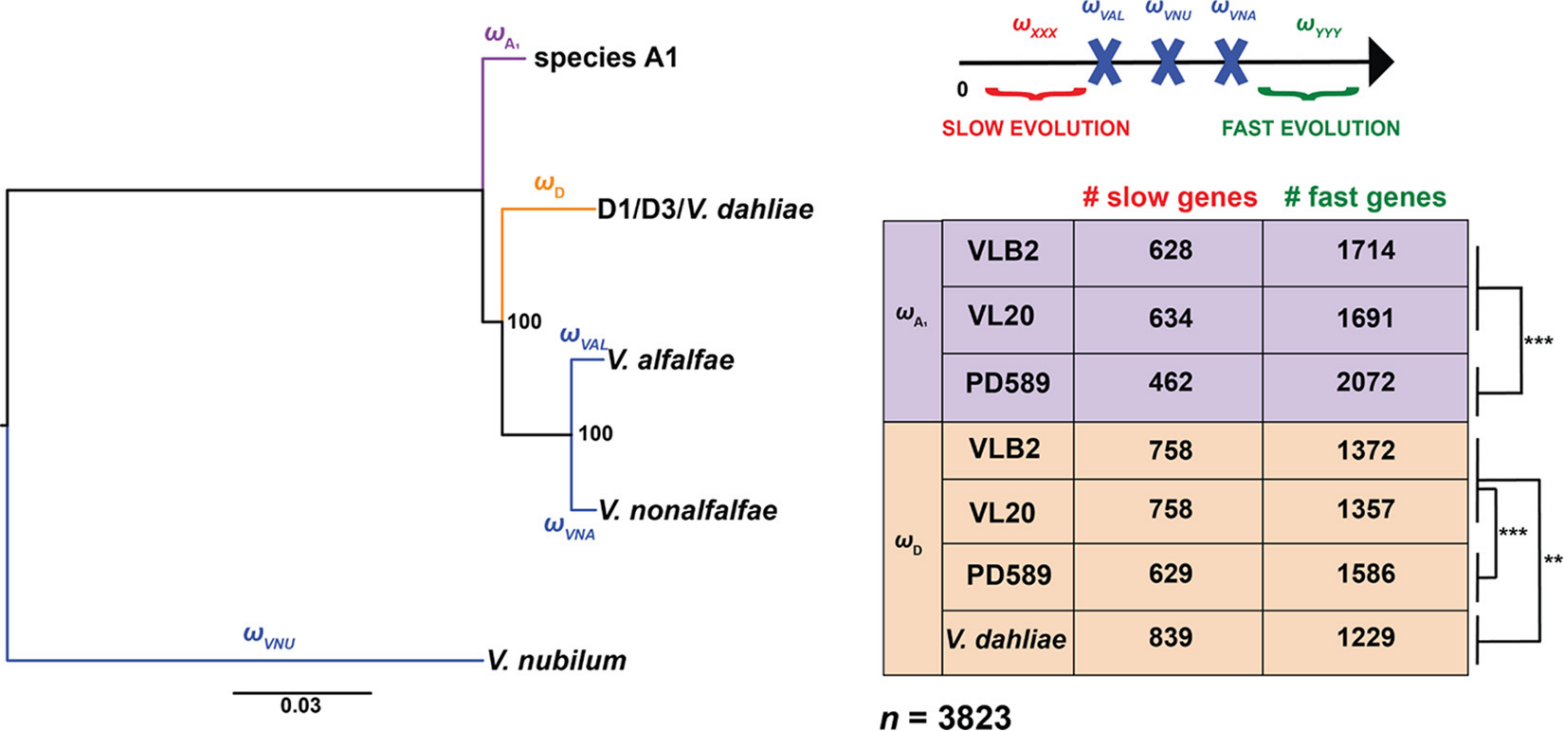
*Verticillium longisporum* genes diverge faster than Verticillium dahliae orthologs. *K_a_*/*K_s_* ratios (ω) were calculated for the tree branches leading to *Verticillium* spp. of the clade Flavnonexudans genomes and the *V. longisporum* subgenomes. A total of 3,823 genes with one ortholog in all respective *Verticillium* (sub)genomes were analyzed. *V. longisporum* and V. dahliae genes with fast or slow evolution have a higher ω or lower ω, respectively, than their *V. alfalfae* (VAL) *V. nonalfalfae* (VNA), and *V. nubilum* (VNU) orthologs. Significance in gene numbers was calculated with Fisher’s exact test. **, *P < *0.01; ***, *P < *0.001.

To investigate gene sequence evolution after hybridization, we compared the ratios of nonsynonymous (*K_a_*) and synonymous (*K_s_*) substitutions (ω) for branches leading to *Verticillium* species (Fig. 4). To exclude the putative impact of the (partial) chromosome 13 duplication in PD589, we excluded genes of this chromosome from the analysis. Substitution rates were determined for a total of 3,823 genes that have just one ortholog in the analyzed *Verticillium* species, *V. alfalfae*, V. dahliae, *V. nonalfalfae*, and V. nubilum, as well as in each of the *V. longisporum* subgenomes. To mitigate possible biases of different divergence times between the *Verticillium* species, we performed the analyses four times: three times with the two subgenomes of *V. longisporum* strains VLB2, VL20, and PD589 and once with V. dahliae and the A1 subgenome of VLB2 (Fig. 4). *V. longisporum* and V. dahliae genes with higher ω values than their *V. alfalfae*, *V. nonalfalfae*, and *V. nubilum* orthologs were considered quickly evolving, whereas those with lower ω values were considered slowly evolving. Comparing the D1/D3/V. dahliae branch, V. dahliae has 839 slowly evolving genes, which is a higher number than the 758 and 629 slowly evolving genes of the *V. longisporum* D1 and D3 subgenomes, respectively. Conversely, V. dahliae has 1,229 quickly evolving genes, which is lower than the number found for the *V. longisporum* D1 and D3 subgenomes, 1,357/1,372 (VL20/VLB2) and 1,586, respectively (Fig. 4). This observation fits the prevailing hypothesis that hybridization accompanied by genome duplication has a “relaxing” effect on gene evolution (32, 51). Furthermore, the lower number of slowly evolving genes and the higher number of quickly evolving genes in the D3 subgenome are significantly different from those of the D1 subgenome (*P < *0.001 by Fisher’s exact test). Similar to the D subgenomes, the A1 subgenome of lineage A1/D3 has a higher number of quickly evolving genes (2,072 versus 1,691 to 1,714) and a lower number of slowly evolving genes (462 versus 628 to 634) than the A1 subgenome of lineage A1/D1. In conclusion, *V. longisporum* lineage A1/D3 genes generally evolve faster than lineage A1/D1 genes in both subgenomes. This may indicate that A1/D3 evolved for a longer time under the more relaxed gene evolutionary conditions than A1/D1; i.e., A1 and D3 hybridized a longer time ago than A1/D1.

To see whether particular genes evolve faster, we functionally characterized the *V. longisporum* A1/D3 genes that have a higher ω value than their *V. alfalfae*, *V. nonalfalfae*, and *V. nubilum* orthologs but also a higher value than their lineage A1/D1 homologs from the corresponding A1 and D subgenomes to select genes that quickly evolved after the A1 and D1/D3 last common ancestor. In total, 1,350 of the 3,823 (35.3%) analyzed genes were quickly evolving in the PD589 A1 subgenome, and 1,084 (28.4%) were quickly evolving in the D3 subgenome. We screened for GO term, COG, and secreted protein enrichments in these fast-evolving A1/D3 genes, and no enrichments for the COGs and genes encoding secreted proteins were found. In the A1 subgenome, 3 GO terms with a molecular function were significantly enriched, associated with molecule binding (protein and ATP) and ATPase activity. In the D3 subgenome, “ATP binding” was the only significantly enriched GO term, which was also enriched in the A1 subgenome. In conclusion, the more pronounced “gene relaxation” in the A1/D3 lineage than in the A1/D1 lineage does not clearly seem to affect genes with particular functions.

### Expression pattern homogenization in the hybridization aftermath.

**FIG 5:**
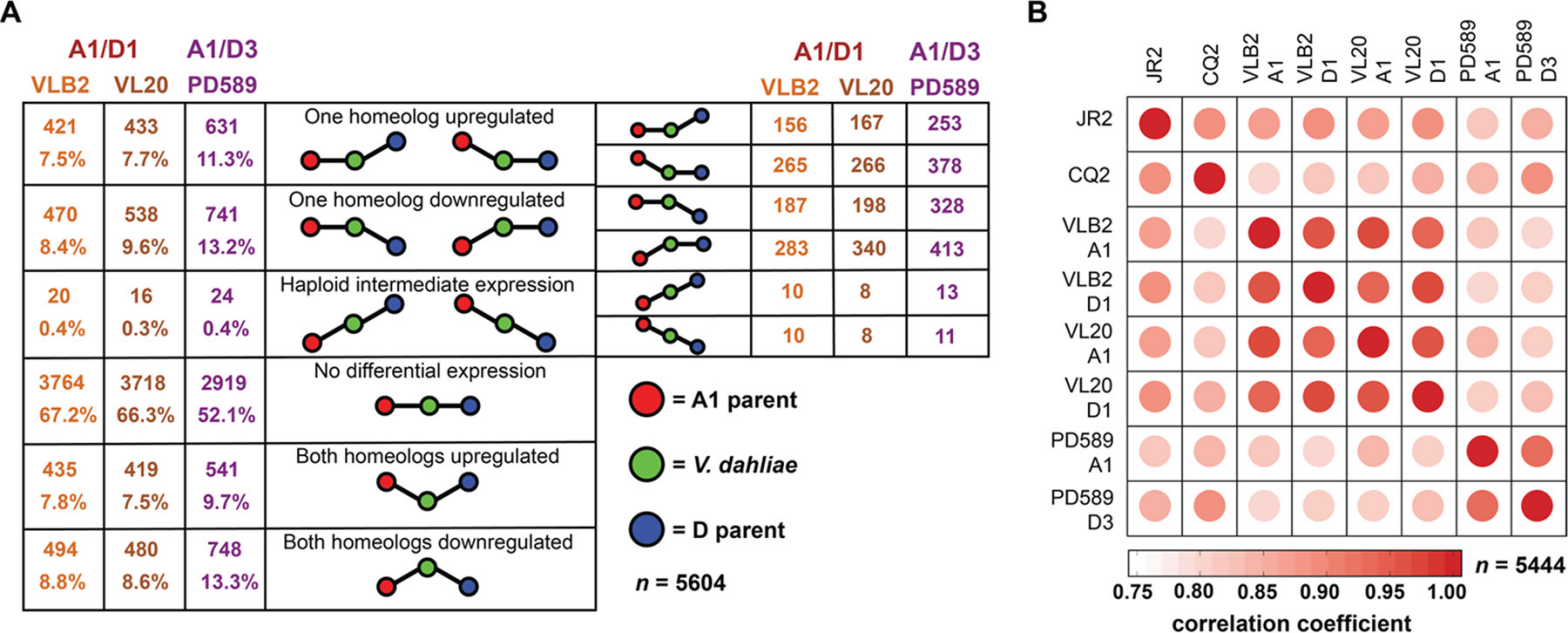
Gene expression patterns of *Verticillium longisporum* subgenomes display a remarkable resemblance. An expression pattern comparison between *Verticillium longisporum* subgenomes and Verticillium dahliae in culture medium was performed. (A) Differential expression between *V. longisporum* and V. dahliae genes. Only genes with one homolog in V. dahliae and two homeologs in *V. longisporum* strains VLB2, VL20, and PD589 were considered for differential expression. The significance of differential expression was calculated using *t* tests relative to a threshold of a log_2_ fold change of 1 and a Benjamini-Hochberg-corrected *P* value cutoff of 0.05. (B) Expression pattern correlation between *V. longisporum* and V. dahliae. Only genes with one homolog in V. dahliae strains JR2 and CQ2 and two homeologs in *V. longisporum* strains VLB2, VL20, and PD589 were considered. Spearman’s correlation coefficients (ρ) were calculated based on the mean transcript-per-million values from three replicates.

To investigate the impact of hybridization on gene expression, the expression of *V. longisporum* genes was compared with that of V. dahliae orthologs from strains grown *in vitro* in potato dextrose broth (PDB). To this end, the expression of single-copy V. dahliae genes was compared with that of orthologs that are present in two homeologous copies in three *V. longisporum* strains (VLB2, VL20, and PD589). Genes on chromosome 13 from strain PD589 and their homologs were excluded from the analysis to avoid putative biases due to a (partial) chromosome duplication, and in total, 5,604 expressed genes were compared. RNA sequencing reads were mapped to the predicted *V. longisporum* genes, of which 50 to 51% mapped to species A1 homeologs and 49 to 50% mapped to the D homeologs. Thus, we observed no global differences in overall contributions to gene expression of the subgenomes. Over half of the *V. longisporum* homeologs display no differential expression with their V. dahliae orthologs, indicating that the majority of the genes did not evolve differential expression patterns (Fig. 5A). In both lineages, higher numbers of differently expressed genes were found in the A1 subgenome than in the D subgenomes: 27 versus 23% for A1/D1 and 38 versus 34% for A1/D3, respectively. The higher fraction of differentially expressed A1 genes is in accordance with the more distant phylogenetic relationship of parent A1 with V. dahliae than of the D parents (Fig. S5). Intriguingly, although D3 diverged more recently from V. dahliae than D1, D3 has more differentially expressed orthologs with V. dahliae than D1. When comparing expression patterns between subgenomes, 11 to 13% of the genes display differential expression between their A1 and D homeologs. Intriguingly, this is more than half the number of differentially expressed D and V. dahliae orthologs (23 to 34%) despite the fact that the D parents diverged more recently from V. dahliae than from species A1 (Fig. S5). In general, the gene expression patterns of the A1 and D subgenomes of the same hybridization event are highly correlated (0.93 to 0.96), higher than D subgenomes and V. dahliae strain JR2 (0.85 to 0.89) and higher than the A1 subgenomes between hybridization events (0.82 to 0.84) (Fig. 5B; Table S2). To compare these expression patterns with the gene expression variation between different V. dahliae strains, we sequenced RNA from cotton-infecting V. dahliae strain CQ2 grown in potato dextrose broth. Although JR2 and CQ2 belong to the same species, their overall gene expression patterns are more dissimilar (ρ = 0.89) than that of *V. longisporum* subgenomes (Fig. 5B; Table S2). The overall discrepancy in the phylogenetic relationship and expression pattern similarities suggests that the subgenome expression patterns of the subgenomes in *V. longisporum* homogenized upon hybridization.

10.1128/mBio.01496-21.9 TABLE S2 Expression pattern correlation of genes between Verticillium dahliae and *Verticillium longisporum* subgenomes grown in culture medium. Download Table S2, DOC file, 0.04 MB. Copyright © 2021 Depotter et al. 2021 Depotter et al. https://creativecommons.org/licenses/by/4.0/ This content is distributed under the terms of the Creative Commons Attribution 4.0 International license.

### Differential homeolog expression occurs in particular gene categories.

**FIG 6:**
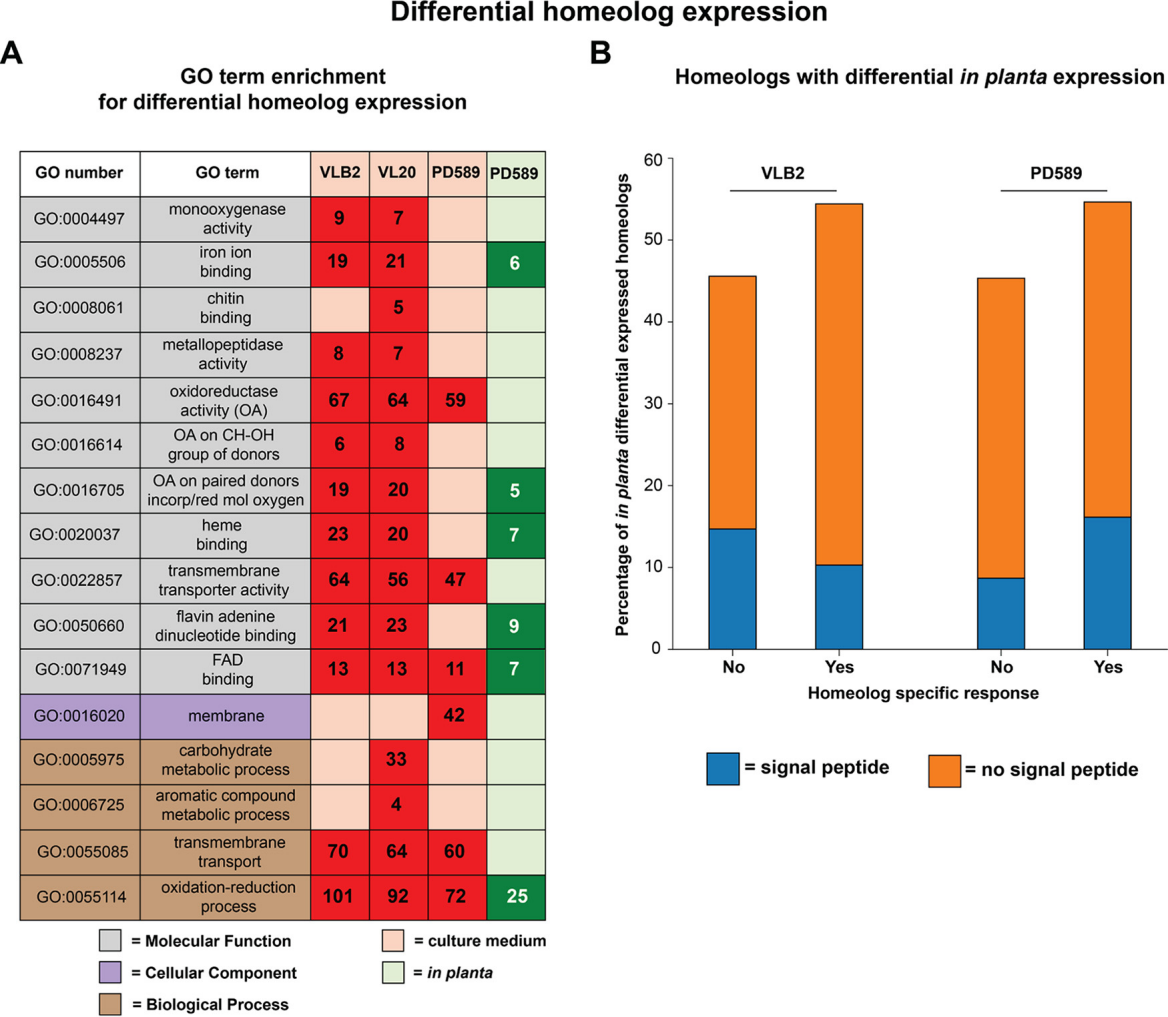
*Verticillium longisporum* displays subgenome-specific gene expression responses. Functional enrichments for *Verticillium longisporum* genes with differential homeolog expression in culture medium and *in planta* are shown. Only *V. longisporum* genes with two homeologs were considered. (A) Gene Ontology (GO) terms that are significantly enriched in differentially expressed homeologs of VLB2, VL20, and PD589. A more detailed overview and levels of significance are reported in Table S3 in the supplemental material. The numbers of genes with differential homeolog expression are indicated. (B) Fractions of genes with differential homeolog expression *in planta* with and without a homeolog-specific response. Genes have a homeolog-specific response if they display differential homeolog expression *in planta* and have no differential or the opposite expression ratio for *V. longisporum* grown in culture medium.

Although parental gene expression patterns appear to have globally homogenized upon hybridization, differential homeolog expression occurs as well (Fig. 5). To assess if genes with differential homeolog expression belong to specific gene groups, we screened for functional enrichments. In total, 10% of the fast-evolving PD589 genes (defined above) have differential homeolog expression, which is significantly lower than the 12% differential homeolog expression for the remainder of the genes (*P < *0.05 by Fisher’s exact test). In both the A1/D1 and A1/D3 lineages, genes with differential homeolog expression are enriched for GO terms related to oxidation-reduction processes, transmembrane transport, and flavin adenine dinucleotide (FAD) binding (Fig. 6A; Table S3). Additionally, the COGs “carbohydrate transport and metabolism” and “secondary metabolite biosynthesis, transport, and catabolism” (Q) are enriched in both lineages (Table S3). Furthermore, we tested if genes encoding secreted proteins were significantly enriched among the genes with differential homeolog expression. Indeed, 23 and 16% of the genes with differential homeolog expression code for a secreted protein in the lineage A1/D1 isolates and the A1/D3 isolate, respectively, whereas this is 9% of the genes that do not display differential expression among homeologs (VLB2, *P* = 1.23E−32; VL20, *P* = 3.71E−29; PD589, *P* = 1.14E−08 [by Fisher’s exact test]). In conclusion, differential homeolog expression seems to be important for particular gene categories, including categories that can be implicated in plant pathogenicity.

10.1128/mBio.01496-21.10 TABLE S3 Functional enrichment analysis of *Verticillium longisporum* genes with differential homeolog expression. Download Table S3, DOC file, 0.1 MB. Copyright © 2021 Depotter et al. 2021 Depotter et al. https://creativecommons.org/licenses/by/4.0/ This content is distributed under the terms of the Creative Commons Attribution 4.0 International license.

### Homeolog-specific expression responses upon plant colonization.

Considering the plant-pathogenic nature of *V. longisporum* and also that genes encoding secreted proteins, which are often implicated in pathogenicity on host plants, are enriched among the genes with differential homeolog expression, we assessed homeolog-specific gene expression during plant colonization. To this end, oilseed rape plants were inoculated with the *V. longisporum* strains VLB2, VL20, and PD589. As observed previously, oilseed rape plants inoculated with VLB2 and PD589 developed typical *Verticillium* symptoms, including stunted plant growth and leaf chlorosis (52). In contrast, oilseed rape plants inoculated with VL20 did not display any disease symptoms. Consequently, we performed total RNA sequencing for oilseed rape plants inoculated with *V. longisporum* strains VLB2 and PD589. For strain PD589, genes on chromosome 13 and their homeologs were removed from the analysis. To assess the quality of the RNA-Seq data, the expression of the 15 highest *in planta*-induced PD589 genes was verified by real-time PCR. In total, 14 of the 15 tested genes had, similar to the RNA-Seq data, a drastic increase in expression upon plant colonization, showing that the real-time PCR data generally correspond to the RNA-Seq data (Fig. S7). For VLB2 and PD589, 51% of the reads mapped to the A1 subgenome, and 49% mapped to the D subgenome. Thus, similar to *in vitro*-grown *V. longisporum*, we did not observe any global difference in the overall contributions to gene expression of one of the subgenomes *in planta*. In total, 1.1% and 2.7% of the homeologs displayed differential expression *in planta*, which is lower than the 11.3 and 13.4% found for VLB2 and PD589 grown *in vitro*, respectively. Genes with differential homeolog expression *in planta* were not enriched for any GO term in the A1/D1 strain VLB2 (Table S3), whereas in the A1/D3 strain PD589, differentially expressed homeologs were enriched for GO terms associated with oxidation-reduction processes and molecular binding (iron ion, heme, and FAD) (Fig. 6A; Table S3). For A1/D1 and A1/D3, genes with differential homeolog expression were enriched for those encoding secreted proteins: 25% of the differentially expressed homeologs encode secreted proteins, and 8 to 9% of the nondifferentially expressed homeologs encode other proteins (*P < *0.05 by Fisher’s exact test). Thus, similar to *in vitro*-grown *V. longisporum*, differential homeolog expression *in planta* is especially important for genes encoding secreted proteins. In 33% of these secretome genes with differential homeolog expression *in planta*, no Pfam domain could be annotated, which is a feature often observed for effector proteins as they are often examples of biological innovation (53). Of these genes that could be functionally annotated, a carbohydrate-active enzyme (CAZyme) function was annotated in 32% of the cases. The remaining part of the functionally annotated genes with differential homeolog expression included other enzymes such as proteases, lipases, carboxylesterases, and peroxidases. We compared genes with differential homeolog expression *in planta* and *in vitro* to assess potential correlations. Intriguingly, over half (54 to 55%) of the differentially expressed homeologs *in planta* are not differentially expressed in culture medium or have the inverse expression pattern, e.g., A1 > D *in vitro* and A1 < D *in planta* (Fig. 6B). Thus, over half of the genes with differential homeolog expression *in planta* display a homeolog-specific response compared to *in vitro* growth. For VLB2, 19% of these genes with a homeolog-specific response encode secreted proteins, whereas 32% of genes with similar differential homeolog expression *in planta* and *in vitro* encode secreted proteins. The opposite pattern was observed for PD589, i.e., 30% with a homeolog-specific response and 19% with similar differential homeolog expression *in planta* and *in vitro*. However, these differences were not significant (*P > *0.05 by Fisher’s exact test). In conclusion, different growth conditions cause homeolog-specific changes in the majority of the *V. longisporum* genes with differential homeolog expression, which are enriched in genes that encode secreted proteins.

10.1128/mBio.01496-21.7 FIG S7 Validation of RNA-Seq data with real-time PCR. The expression levels of 15 *Verticillium longisporum* PD589 genes with the highest increase in gene expression *in planta* compared with *in vitro* were quantified by real-time PCR. The primers used for real-time PCR and the Pfam domain annotations of the amplified genes are indicated. Gene expression was quantified relative to the expression of *VdGAPDH*. Download FIG S7, PDF file, 0.6 MB. Copyright © 2021 Depotter et al. 2021 Depotter et al. https://creativecommons.org/licenses/by/4.0/ This content is distributed under the terms of the Creative Commons Attribution 4.0 International license.

## DISCUSSION

Hybridization is a powerful evolutionary mechanism that can lead to the emergence of new plant pathogens with distinct features compared with their parents (8, 23). Here, we reveal the transcriptomic plasticity of the hybrid pathogen *V. longisporum* and illustrate the parental allele-specific response to different environmental cues. Differentially expressed *V. longisporum* homeologs are enriched for genes encoding secreted proteins that generally act to facilitate environmental manipulation (54). Interestingly, over half of the differentially expressed homeolog genes *in planta* display different relative contributions *in vitro*. Thus, upon the environmental changes that are associated with different growth conditions, *V. longisporum* encounters subgenome-specific gene expression alterations, leading to differential homeolog expression. Although not previously reported for any other hybrid plant pathogen, subgenome-specific gene expression alterations have previously been reported to occur in the artificial yeast hybrid S. cerevisiae × Saccharomyces uvarum upon a temperature change (55). Genes with these subgenome-specific responses were involved in a variety of biological processes, including the trehalose metabolic process that is involved in thermotolerance. Thus, more generally, hybrid fungi, comprising natural as well as artificial hybrids, respond to environmental changes in an allele-specific manner, especially for genes that manipulate or mitigate environmental changes. Secretome genes with differential homeolog expression *in planta* often have an enzymatic function or lack an annotated Pfam domain, which is a feature often observed for effector proteins that act in pathogenicity (53). Thus, conceivably, homeolog-specific responses *in planta* occur in genes that are important for host colonization. Similarly, differential homeolog expression in the hybrid opportunistic human pathogen Candida orthopsilosis involves genes that are implicated in host interactions, related to superoxide dismutase activity and zinc metabolism (56).

Although differential homeolog expression occurs, the general tendency is that expression patterns between the A1 and D subgenomes homogenized upon hybridization (Fig. 5). Despite the absence of A1 and D1 species due to their enigmatic nature, we can conclude that parental gene expression patterns homogenized in the aftermath of hybridization as subgenome expression patterns display more resemblance than the expression pattern between V. dahliae and the D parents and between the A1 subgenomes of different hybridization events (Fig. 5B; see also Table S2 in the supplemental material). Homogenization of parental expression patterns has been similarly observed in the fungal allopolyploid *Epichloë* strain Lp1 (36) as well as in the artificial hybrid S. cerevisiae × S. uvarum, where the extent of differential ortholog expression between the parents was diminished upon hybridization (57). Thus, gene expression homogenization seems to be a more general phenomenon in fungi. Gene expression divergences may evolve through mutations in regulatory sequences of the gene itself (*cis*-effects), such as promoter elements, or alterations in other regulatory factors (*trans*-effects), such as chromatin regulation (58, 59). Conceivably, the higher correlation in homeolog expression patterns than in parental ortholog expression patterns originates from changes in *trans* regulators, as homeologs, in contrast to orthologs, share the same nuclear environment (59). Intriguingly, parent D3 has more genes that are differentially expressed with V. dahliae orthologs than parent D1, even though D3 diverged more recently from V. dahliae than D1 (Fig. 5; Fig. S5). Correspondingly, the A1 subgenome of lineage A1/D3 displays more differential gene expression with V. dahliae than the A1 subgenome of the A1/D1 lineage. This may indicate that A1 and D3 hybridized before A1 and D1, as more distinct expression patterns may have evolved over time.

In addition to the transcriptomic plasticity of homeolog expression upon environmental changes, *V. longisporum* is also plastic on a genomic level, which is displayed by its mosaic structure (Fig. 1A; Table S1). Mosaicism is also observed in the grass pathogen Zymoseptoria pseudotritici, which is a close relative of the prominent wheat pathogen Zymoseptoria tritici (29). Z. pseudotritici is a homoploid hybrid that displays mosaicism on a population level where genome regions inherited from one parent display low variation, whereas highly variable genome regions were transmitted from both parents. *V. longisporum* mosaicism is caused by extensive genomic rearrangements after hybridization (Fig. 2B and Fig. 3). Genomic rearrangements are major drivers of evolution and facilitate adaptation to novel or changing environments (49). Genomic rearrangements are not specific to the hybrid nature of *V. longisporum* as other *Verticillium* species similarly encountered extensive chromosomal reshuffling (44, 45, 50, 60). In V. dahliae, genomic rearrangements especially occur in genomic regions that were originally described as lineage-specific regions, which are enriched for active transposable elements, and which are derived from segmental duplications that were followed by extensive reciprocal gene losses, encounter nucleotide sequence conservation, and have a unique epigenomic profile (50, 60–63). These lineage-specific regions are enriched for *in planta*-expressed genes and contain effector genes that facilitate host infection (60, 61, 64, 65). More recently, these lineage-specific regions have been referred to as dynamic chromosomal regions (61). Similar to V. dahliae, syntenic breaks in *V. longisporum* often reside in repeat-rich genome regions, as repetitive sequences (Fig. S6), due to their abundance, are more likely to act as a substrate for the unfaithful repair of double-strand DNA breaks (49, 50). However, the presence of two genomes within a single hybrid nucleus may also provide homeologous sequences with sufficient identity to mediate unfaithful repair.

The *V. longisporum* D genomes globally display accelerated evolution compared with their V. dahliae orthologs (Fig. 4), which may be a consequence of genome doubling. Interestingly, the *V. longisporum* A1/D3 lineage strain PD589 encountered more divergent gene evolution than the A1/D1 lineage strains VLB2 and VL20 in both subgenomes, indicating that the A1/D3 hybridization event occurred prior to the A1/D1 hybridization event as a longer allodiploid state could facilitate extended sequence divergence (66). However, accelerated evolution is not consistently observed in fungi as deceleration upon allopolyploidization has been recorded in the fungal genus *Trichosporon* (67). Arguably, environmental cues play an important role in the speed and grade of gene diversification upon allopolyploidization (68). Possibly, accelerated gene evolution in *V. longisporum* is cued by a host range alteration, as it is, in contrast to haploid *Verticillium* species, a Brassicaceae specialist (42). However, we did not find functional enrichments in fast-evolving genes that point toward that hypothesis. Moreover, as the A1 species remains enigmatic, we cannot be sure that a host shift occurred (39, 41).

Whole-genome duplication events are typically followed by extensive gene loss, often leading to reversion to the original ploidy state (69). For instance, the artificial interspecific hybrid S. cerevisiae × S. uvarum encountered nine independent events where loss of heterozygosity occurred after evolving for hundreds of generations under nutrient-limited conditions (70). Heterozygosity loss has proceeded to only a limited extent in *V. longisporum*, as 84% of lineage A1/D1 genes and 79% of lineage A1/D3 genes are present in two copies, whereas the haploid V. dahliae contains only 0.4% of its genes in two copies (Fig. 2B). Thus, the *V. longisporum* genome displays the symptoms of a recent allodiploid, with gene loss being an ongoing process that by now has progressed only marginally. Heterozygosity loss can indicate deleterious epistatic interactions between parental genomes that need to homogenize in order for the hybrid to be viable. Similar to other fungal hybrids (70, 71), we did not observe a specific group of genes where loss of heterozygosity was selected for. The degree of haploidization is a third indication that the A1/D3 lineage likely hybridized prior to A1/D1, as haploidization progressed further in A1/D3 than in A1/D1 (Fig. 2B). C. orthopsilosis hybrids from different hybridization events have different degrees of heterozygosity loss, but genes where homeologs are maintained in both hybrids are enriched for those that have differential homeolog expression (56). Although species often revert to their original ploidy state after polyploidization, the retention of both homeolog copies can also be evolutionarily advantageous, for instance, to respond in a parental allele-specific fashion to environmental cues (Fig. 6).

### Conclusion.

Allodiploidization is an intrusive evolutionary mechanism in fungi where two chromosome sets from parents with distinct evolutionary histories merge. Consequently, most genes obtain an additional gene copy that can be differentially regulated according to the environmental conditions. Thus, allodiploid fungi can respond in a parental allele-specific fashion to environmental cues. Besides such parental allele-specific gene expression, allodiploidization furthermore contributed to dynamic genome evolution through rearrangements between parental chromosome sets and accelerated gene evolution in *V. longisporum*. Thus, in comparison to haploid *Verticillium* species, *V. longisporum* has high adaptive potential that can contribute to host immunity evasion and may explain its specialization toward brassicaceous plant hosts.

## MATERIALS AND METHODS

### *V. longisporum* genome sequencing and assembly.

Genome assemblies of *V. longisporum* strains VLB2 and VL20 were previously constructed using long reads obtained through single-molecule real-time (SMRT) sequencing (40). Here, we sequenced *V. longisporum* strain PD589 using Oxford Nanopore technology. In order to obtain DNA of PD589, spores were harvested from potato dextrose agar (PDA) plates and grown in 1/5 potato dextrose broth (PDB) for 5 days. Mycelium and spores were collected on Microcloth, freeze-dried overnight, and ground to a fine powder. For DNA isolation, 100 mg of material was used and incubated for 1 h at 65°C with 800 μl DNA extraction buffer (0.35 M sorbitol, 0.1 M Tris base, 5 mM EDTA [pH 7.5]), nucleic lysis buffer (0.2 M Tris, 0.05 M EDTA, 2 M NaCl, 2% cetyltrimethylammonium bromide [CTAB]), and Sarkosyl (10%, wt/vol) in a 2:2:1 ratio. Subsequently, a 1/2 volume of phenol-chloroform-isoamyl alcohol (25:24:1) was added, and the mixture was shaken vigorously and incubated at room temperature (RT) for 5 min before centrifugation at maximum speed (16,000 rpm) for 15 min (RT). The upper (aqueous phase) layer was transferred to a new tube, 5 μl of RNase (10 mg/μl) was added, and the mixture was incubated at 37°C for 1 h. Next, a 1/2 volume of chloroform was added, mixed, and centrifuged at maximum speed for 10 min at RT. The upper layer was transferred to a new tube, and a second chloroform wash step was performed. After transferring the upper layer to a new tube, it was mixed with 1 volume (∼800 μl) of 100% ice-cold ethanol by gently inverting the tube, and finally, the DNA was fished out and washed twice by applying 500 μl of 70% ethanol. Finally, the DNA was air dried, resuspended in nuclease-free water, and stored at 4°C overnight. The DNA quality, size, and quantity were assessed by nanodrop, gel electrophoresis, and Qubit analyses, respectively.

To sequence *V. longisporum* strain PD589 DNA, a library was prepared according to the manufacturer’s protocol provided by ONT (catalog number SQK-RAD004), with an initial amount of ∼400 ng high-molecular-weight (HMW) DNA. The library was loaded onto an R9.4.1 flow cell, which ran for 24 h and yielded ∼7 Gb of data. ONT sequencing reads were base called using Guppy (version 3.1.5), using the high-accuracy base-calling algorithm. Subsequently, adapter sequences were identified and removed using Porechop (version 0.2.3; default settings), adapters at the end of the reads were trimmed, and reads with internal adapters were discarded. To be able to polish the genome assembly, we used the same HWA DNA isolated for ONT sequencing to generate ∼35 million high-quality (>95%; Phred score of 20) 150-bp paired-end reads (∼76× coverage) using the BGISeq platform (BGI Tech Solutions, Hong Kong, China).

The *V. longisporum* PD589 genome was *de novo* assembled using Canu (version 1.8; genomeSize=70m, corOutCoverage=100, batOptions=‘-dg 3 -db 3 -dr 1 -ca 500 -cp 50’) (72). In total, 924,740 cleaned ONT reads were used for the *de novo* assembly, of which 743,753 were >1 kb (∼88× coverage). The genome assembly was polished using two sequential rounds of Apollo (version 1.1) (73). To this end, the high-quality paired-end reads were mapped to the genome assembly using bwa (version 0.7.17-r1188; default settings) (74).

To improve the assemblies to (nearly) the chromosome level, chromatin conformation capture (Hi-C) followed by high-throughput sequencing was performed for VLB2, VL20, and PD589, using methods similar to the ones previously reported (44). For the three *V. longisporum* strains, 1 million spores were added to 400 ml potato dextrose broth and incubated for 6 days at 22°C with continuous shaking at 120 rpm. A total of 300 mg (fresh weight) mycelium was used as the input for generating Hi-C sequencing libraries with the Proximo Hi-C kit (microbe) (Phase Genomics, Seattle, WA, USA), according to the manufacturer’s instructions. Hi-C sequencing libraries were paired-end (2 by 150 bp) sequenced on the NextSeq500 platform at USEQ (Utrecht, The Netherlands). Juicer (v1.6) was then used to map Hi-C sequencing reads to the previously obtained assemblies (75). The contact matrices generated by Juicer were used by the three-dimensional (3D) *de novo* assembly (3D-DNA) pipeline (v180922) to eliminate misjoints in the previous assemblies (76). The assemblies were manually further improved using Juicebox Assembly Tools (JBAT) (v.1.11.08) (77). JBAT was subsequently used to determine the centromere location based on intra- and interchromosomal contact frequencies. Only contigs that were larger than 100 kb were maintained in the assembly. Coverage of ONT sequencing for the *V. longisporum* PD589 assembly was determined for 20-kb windows with SAMtools depth (v1.9) (78), and reads were mapped with minimap2 (v2.17-r941) (79).

The mitochondrial genomes of the haploid *Verticillium* species were previously sequenced and assembled (45). Mitochondrial *V. longisporum* genomes were assembled alongside the nuclear genomes (40). Mitochondrial contigs consisted of multiple copies of the mitochondrial genome due to its circular nature. A single copy of the mitochondrial genome was excised using BEDTools getfasta (v2.23.0) (102). Filtered *V. longisporum* subreads were mapped to these single-copy mitochondrial assemblies using circlator (v1.5.5) (103). The mapped reads were subsequently used to make a new *V. longisporum* mitochondrial genome assembly using SAMtools mpileup (v1.8) (78).

### RNA sequencing.

To obtain RNA-Seq data for *Verticillium* grown in culture medium, V. dahliae isolates JR2 and CQ2 and *V. longisporum* isolates VLB2, VL20, and PD589 were grown for 3 days in PDB, with three biological replicates for each isolate. To obtain RNA-Seq data from *in planta* growth, 2-week-old plants of the susceptible oilseed rape cultivar ‘Quartz’ were inoculated by dipping the roots for 10 min in a spore suspension of 1 × 10^6^ conidiospores ml^−1^ of *V. longisporum* isolates VLB2, VL20, and PD589 (52). After root inoculation, plants were grown in individual pots in a greenhouse under a cycle of 16 h of light and 8 h of darkness, with temperatures maintained between 20°C and 22°C during the day and at a minimum of 15°C overnight. Three pooled samples (10 plants per sample) of stem fragments (3 cm) were used for total RNA extraction. Total RNA was extracted based on TRIzol RNA extraction (104). cDNA synthesis, library preparation (TruSeq RNA-Seq short-insert library), and Illumina sequencing (single-end 50 bp) were performed at the Beijing Genome Institute (BGI) (Hong Kong, China).

The extracted RNA was reverse transcribed according to the instructions of the Moloney murine leukemia virus (M-MLV) reverse transcriptase protocol (Promega, Madison, WI, USA). The expression of the tested genes was quantified with real-time PCR relative to *VdGAPDH* as previously described (64). The primers used can be found in Fig. S7. The assay was performed for three biological replicates of *V. longisporum* PD589 grown *in planta* and *in vitro*. The log_2_ fold change in gene expression was determined based on the median of the *in planta* and *in vitro* expression levels.

### Gene prediction and functional characterization.

The *V. longisporum* assemblies of strains VLB2, VL20, and PD589 and the previously published assemblies of V. dahliae strains JR2 and CQ2 (47, 62) were annotated using the BRAKER v2.1.4 pipeline for RNA-Seq data with the options “–softmasking” and “–fungus” enabled (48). RNA-Seq reads from *Verticillium* grown in axenic cultures (all replicates) were mapped to the assemblies using TopHat v2.1.1 (80). Predicted genes with internal stop codons, without a start codon, or with an unknown amino acid in the encoded protein sequence were removed from the analysis. The secretome prediction was done using SignalP5 (v5.0) (81). Pfam and Gene Ontology (GO) function domains were predicted using InterProScan (v5.42-78.0) (82). Clusters of Orthologous Group (COG) categories were determined for protein sequences using eggNOG-mapper (v2.0) with the taxonomic scope set on Ascomycota (83, 84). Carbohydrate-active enzymes (CAZymes) were annotated using the dbCAN2 meta server (85, 86). A protein was considered a CAZyme if at least two of the three tools (HMMER, DIAMOND, and Hotpep) predicted a CAZyme function.

### Parental origin determination.

Subgenomes were divided based on the differences in sequence identities between species A1 and D1/D3 with V. dahliae. *V. longisporum* genomes of VLB2, VL20, and PD589 were aligned to the complete genome assembly of V. dahliae JR2 using NUCmer (v3.1), which is part of the MUMmer package (87). Here, only 1-to-1 alignments longer than 10 kb and with a minimum of 80% identity were retained. Subsequent alignments were concatenated if they aligned to the same contig with the same orientation and order as the reference genome so that lineage-specific genomic regions could also be assigned to a subgenome. The average nucleotide identity was determined for every concatenated alignment and used to divide the genomes into subgenomes. In addition, the sequence identities of orthologous coding regions between *V. longisporum* and V. dahliae were determined using the Needle-Wunsch algorithm implemented in EMBOSS (v6.6.0.0) (88). Alignment and coding sequence identities were both used to determine the parental origin of genome regions. Differences in GC content between homologous genes present in two copies were calculated as described previously (28). GC contents of gene coding regions were calculated with infoseq from EMBOSS (v.6.6.0.0) (88). The features to indicate the biparental origin of the *V. longisporum* genomes were visualized using the R package circlize (v.0.4.10) (89).

### Genome analysis.

The quality of genome assemblies was assessed by screening for the presence of benchmarking universal single-copy orthologs (BUSCOs) using BUSCO software version 4.0.6 with the database “ascomycota_odb10” (90).

Repeats were *de novo* identified using RepeatModeler (v1.0.11) and combined with the repeat library from RepBase (release 20170127) (91). The genomic location of repeats was identified with RepeatMasker (v4.0.6).

The phylogenetic relationship of the nuclear and mitochondrial (sub)genomes of the *Verticillium* species of the clade Flavnonexudans (38) was determined using the following haploid strains: *V. alfalfae* PD683, V. dahliae JR2, *V. nonalfalfae* TAB2, and *V. nubilum* PD621 (45, 47). Phylogenetic trees based on nuclear DNA were constructed based on the ascomycete BUSCOs that were shared by all the included species (90). Nucleotide sequences were separately aligned using MAFFT (v7.464) (92). Phylogenetic trees were inferred using RAxML with the Generalised Time Reversible (GTR)+GAMMA substitution model (v8.2.11) (93). The robustness of the inferred phylogeny was assessed by 100 rapid bootstrap approximations.

Homologs in *Verticillium* were determined using nucleotide BLAST (v2.2.31+). Genes with a minimum identity of 80% and a minimum overlap of 80% were considered homologs, which were determined using SiLiX (v.1.2.10-p1) software (94).

Global nucleotide alignments using the Needle-Wunsch algorithm of the EMBOSS package were used to determine homologous gene pairs in VLB2 and VL20 (v6.6.0.0) (88). Sequence identities between these genes in copy were determined based on their global alignment. Synteny between the VLB2 and VL20 genome assemblies was determined by using one-to-one alignments obtained with NUCmer (v3.1), which is part of the MUMmer package (87). Synteny was visualized with the R package circlize (v.0.4.10) (89).

Gene clusters were identified using CROC with a minimum number of 3 genes that can be found for a cluster using a gene window of 6 (95). Here, the Benjamini-Hochberg method was used to correct for multiple testing.

### Gene divergence.

Previously published annotations of the haploid *Verticillium* species V. dahliae, *V. alfalfae*, *V. nonalfalfae*, *V. nubilum*, V. tricorpus, and *V. albo-atrum* were used to compare the evolutionary speeds of orthologs (45, 47). VESPA (v1.0b) software was used to automate this process (96). The coding sequences for each *Verticillium* species were filtered and subsequently translated using the VESPA “clean” and “translate” functions. Homologous genes were retrieved by protein BLAST (v2.2.31+) querying a database consisting of all *Verticillium* protein sequences. Here, the options “-max_hsps 1” and “-qcov_hsp_perc 80” were used. Homologous genes were grouped with the VESPA “best_reciprocal_group” function. Only homology groups that comprised a single representative for every *Verticillium* species were used for further analysis. Protein sequences of each homology group were aligned with muscle (v3.8) (97). The aligned protein sequences of the homology groups were converted to nucleotide sequences by the VESPA “map_alignments” function. The alignments were used to calculate the *K_a_*/*K_s_* ratio for every branch of the species phylogeny using the codeml module of PAML (v4.9) with the following parameters: F3X4 codon frequency model, wag.dat empirical amino acid substitution model, and no molecular clock (98). To this end, the following phylogenetic tree topology was used: ((((V. dahliae/D1/D3,(*V. alfalfae*, *V. nonalfalfae*)),A1),*V. nubilum*),(*V. tricorpus*, *V. albo-atrum*)). Divergence was compared only for genes that are present in the two subgenomes of *V. longisporum* strains VLB2, VL20, and PD589.

### Gene expression analysis.

The RNA sequencing reads were filtered using the Trinity software (v2.9.1) option Trimmomatic under standard settings (99). The reads were then mapped to the *Verticillium* genomes using Bowtie 2 (v2.3.5.1), with the first 15 nucleotides on the 5′ end of the reads being trimmed because of inferior quality (100). To compare gene expression patterns, homologs were retrieved by nucleotide BLAST (v2.2.31+). Genes with a minimum identity of 80% and a minimum overlap of 80% were considered homologs, which were determined using SiLiX (v.1.2.10-p1) software (94). Reads were counted for the predicted gene coding regions using the R package Rsubread (v1.34.7). Significant differential expression of a locus was calculated using the R package edgeR (v3.26.8) (101). The significance of differential expression was calculated using *t* tests relative to a threshold of a log_2_ fold change of 1 with Benjamini-Hochberg correction using a *P* value cutoff of 0.05.

### Data availability.

Raw RNA-Seq reads and genome assemblies have been deposited at the NCBI database under the BioProject accession number PRJNA473305.
