# Supplementary figures and images for: The Interspecific Fungal Hybrid Verticillium longisporum Displays Subgenome-Specific Gene Expression
Source: mBio. 2021 Jul 20;12(4):e01496-21. doi: 10.1128/mBio.01496-21 (PMC8406199; doi:10.1128/mBio.01496-21)

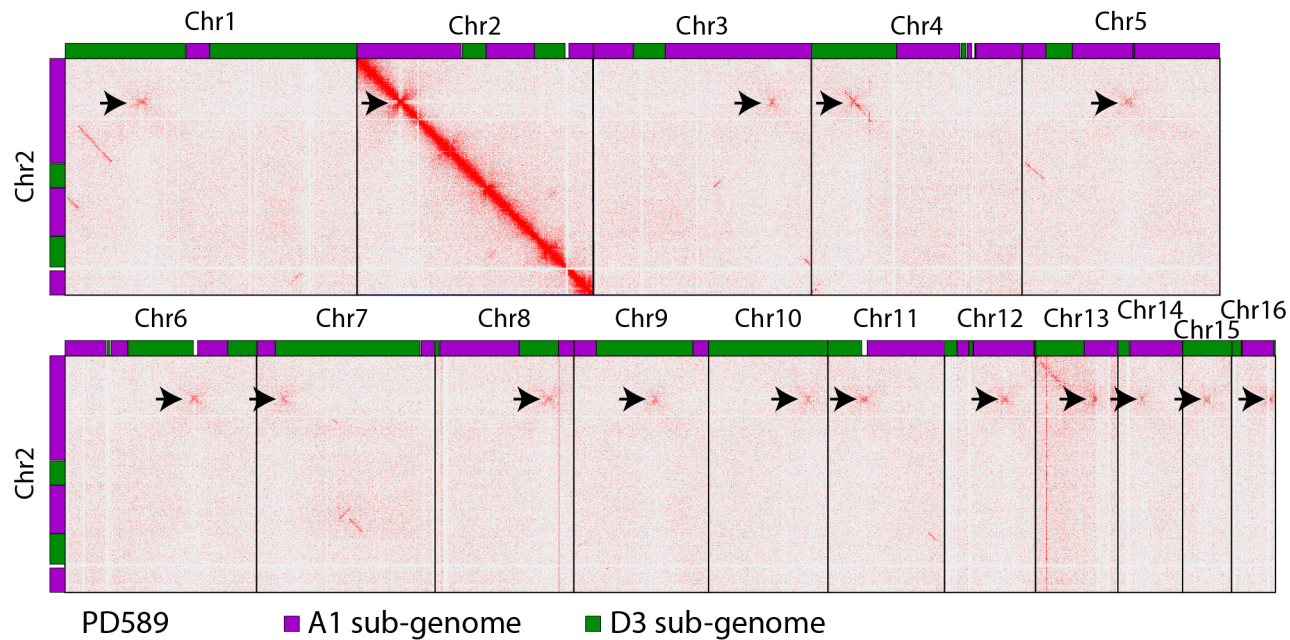

Supplement: FIG S1 [file mbio.01496-21-sf001.pdf]

**A**

## Genome regions

**VLB2**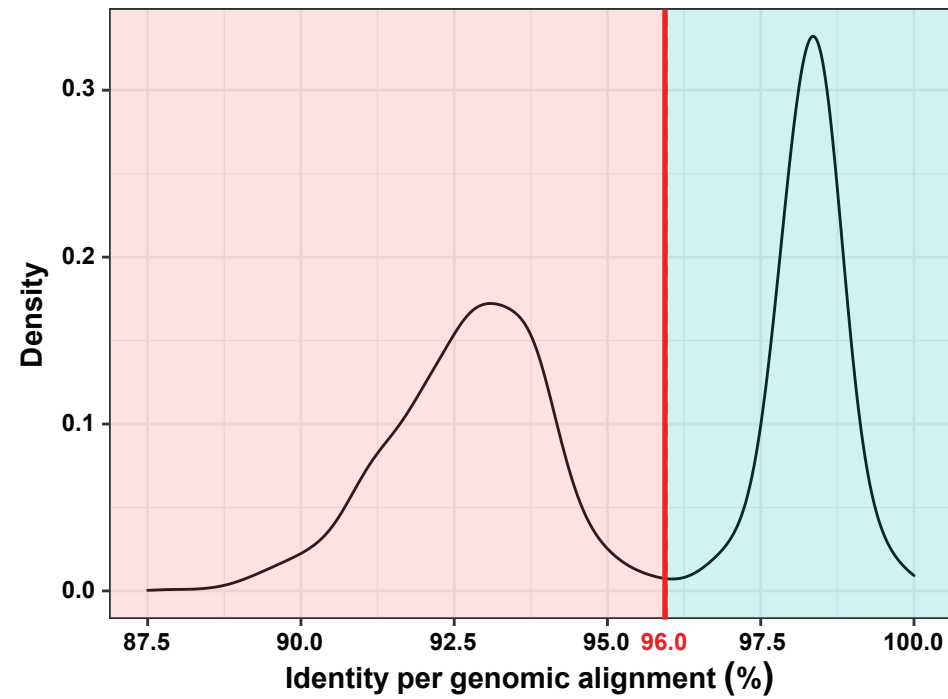**VL20**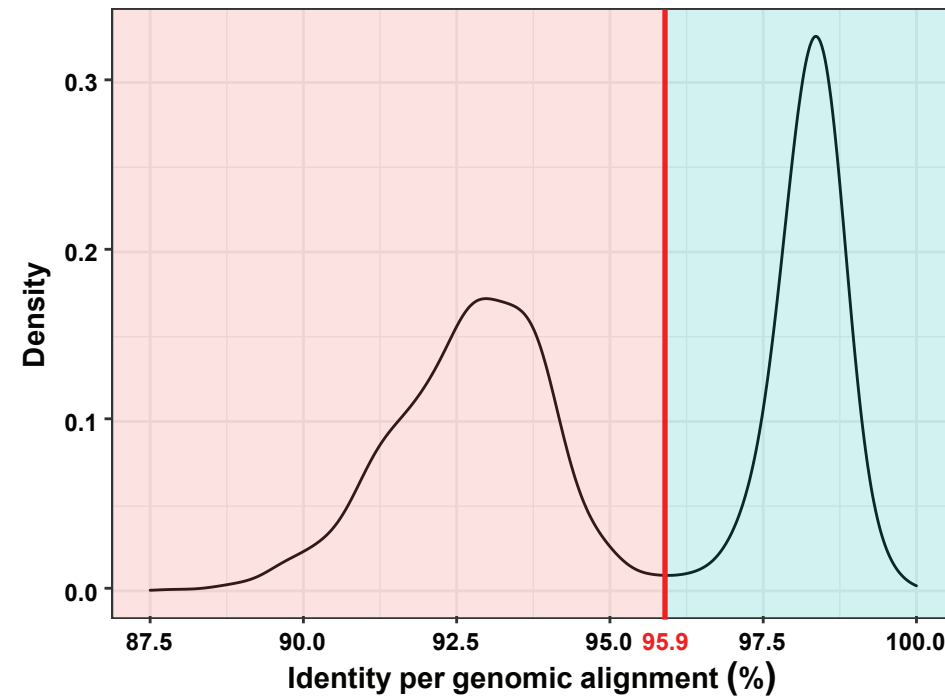**PD589**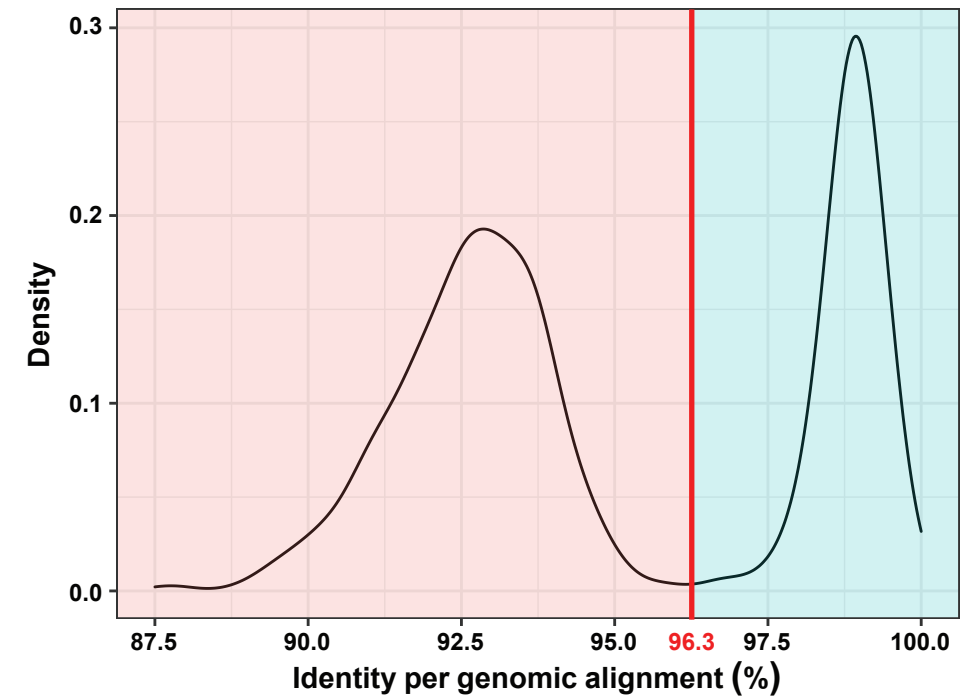**B**

## Coding sequence

**VLB2**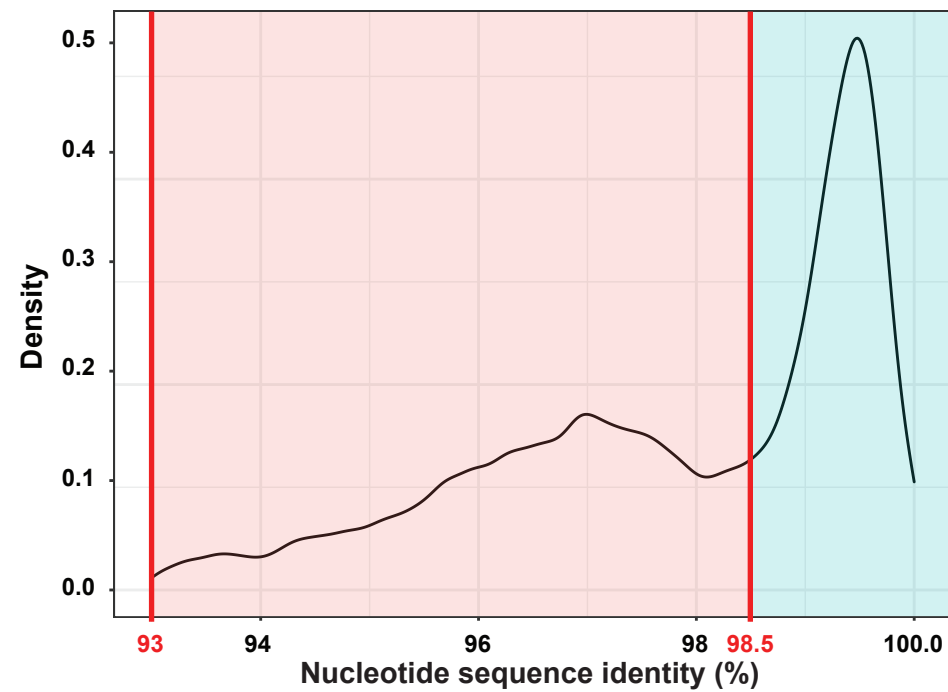**VL20**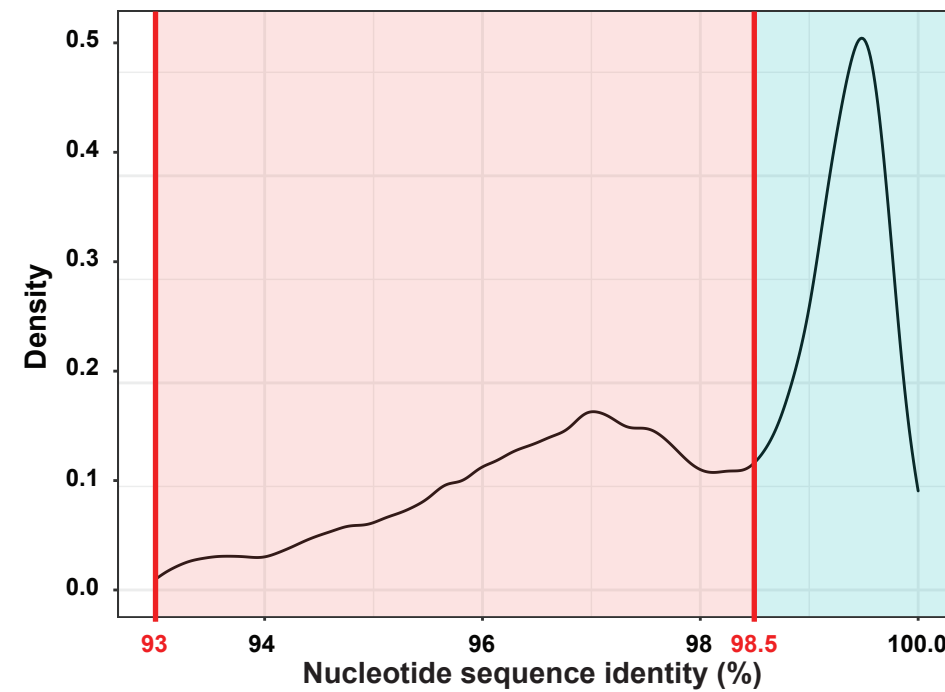**PD589**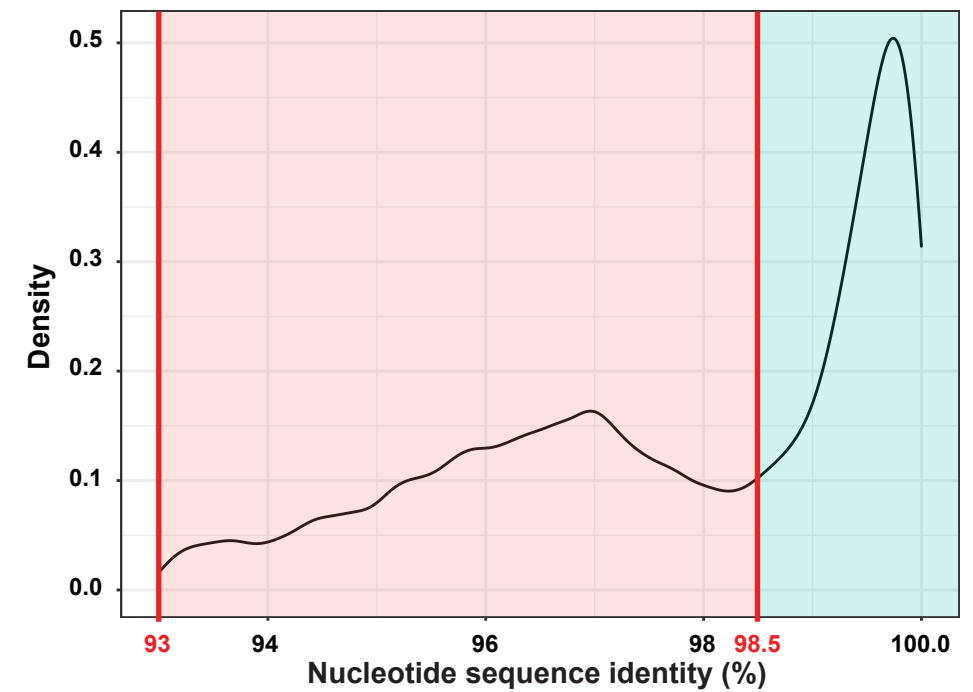

Supplement: FIG S3 [file mbio.01496-21-sf003.pdf]

■ = A1 sub-genome    ■ = D1 sub-genome    ■ = Undetermined

VLB2

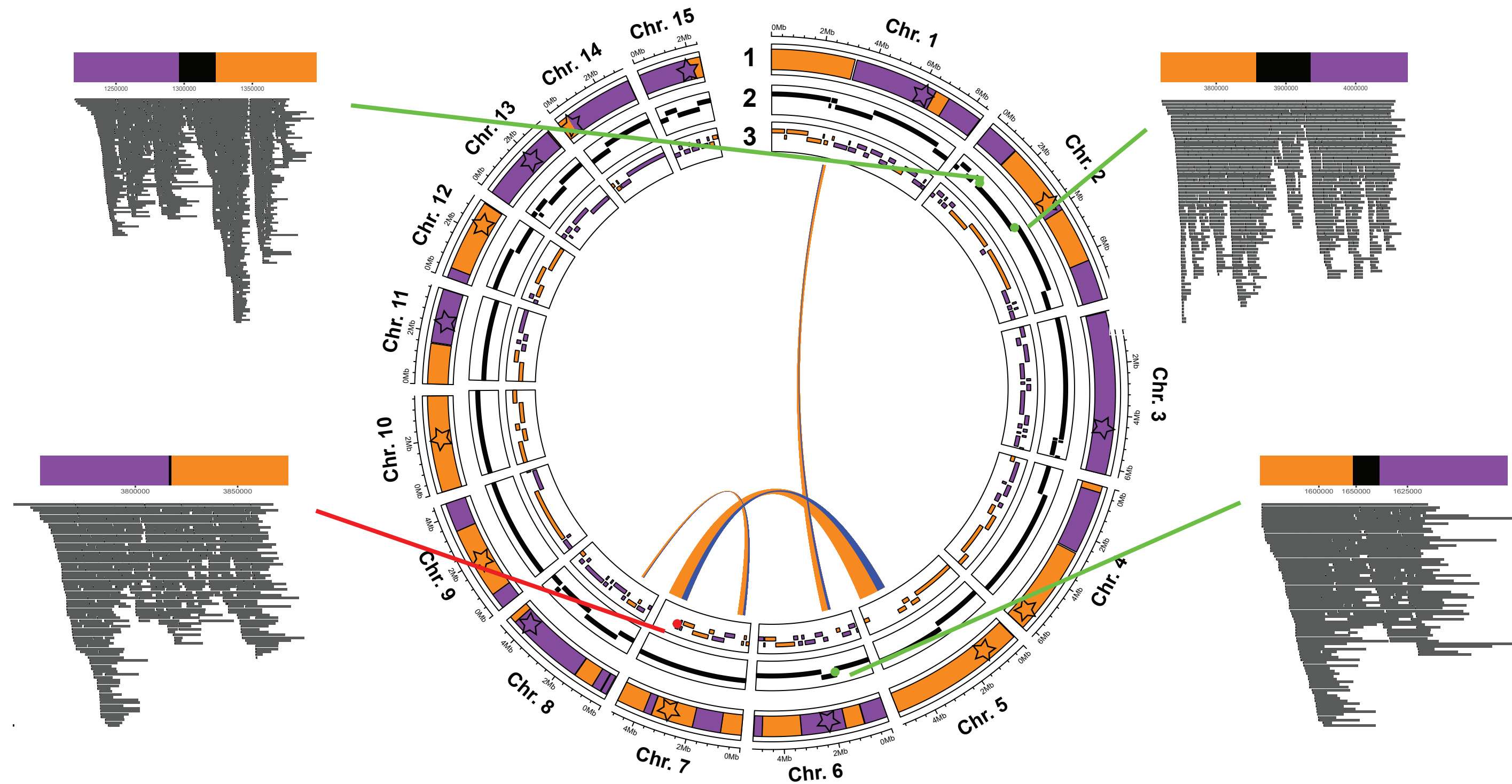

VL20

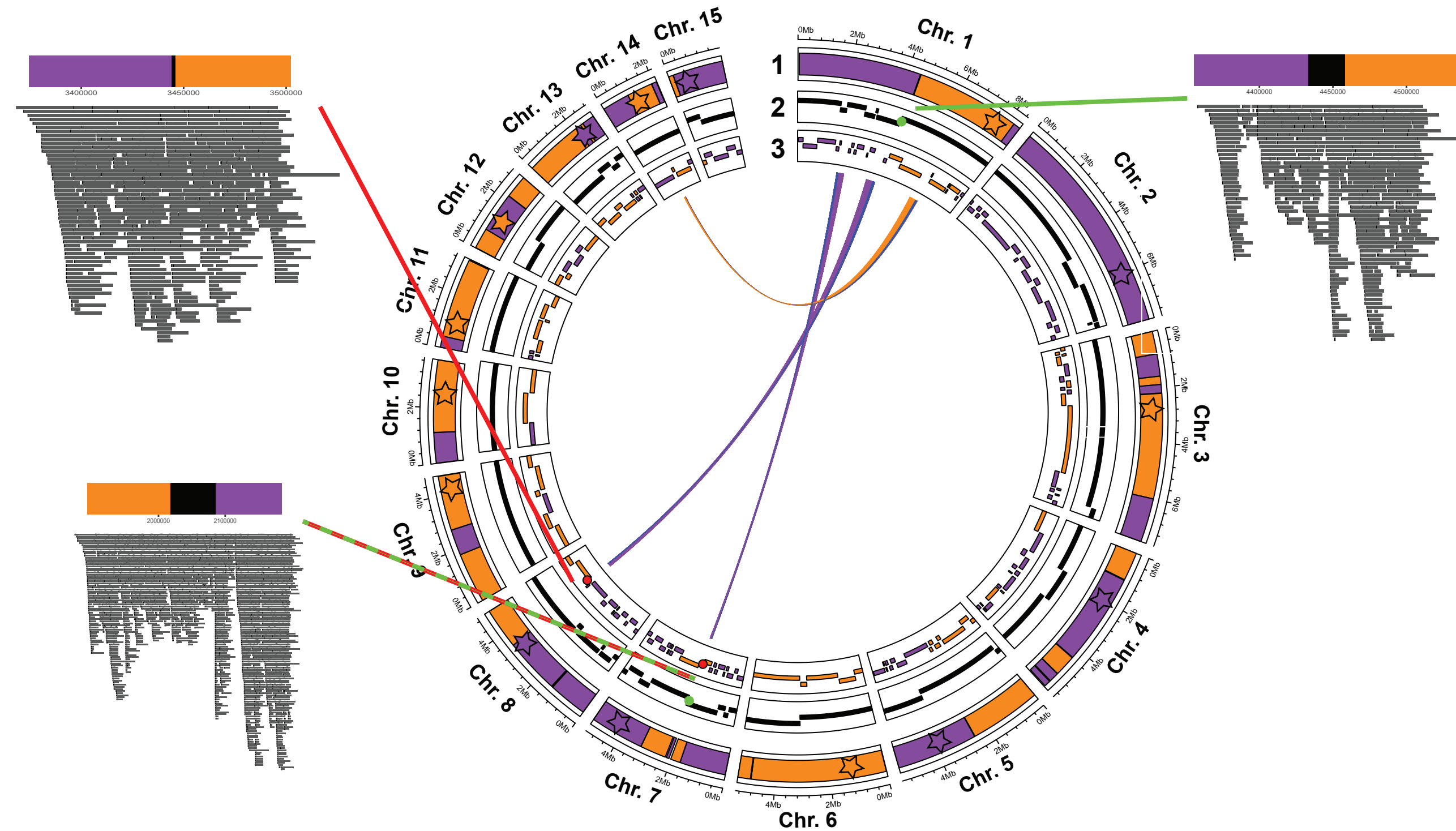

Supplement: FIG S4 [file mbio.01496-21-sf004.pdf]

## Nuclear genomes

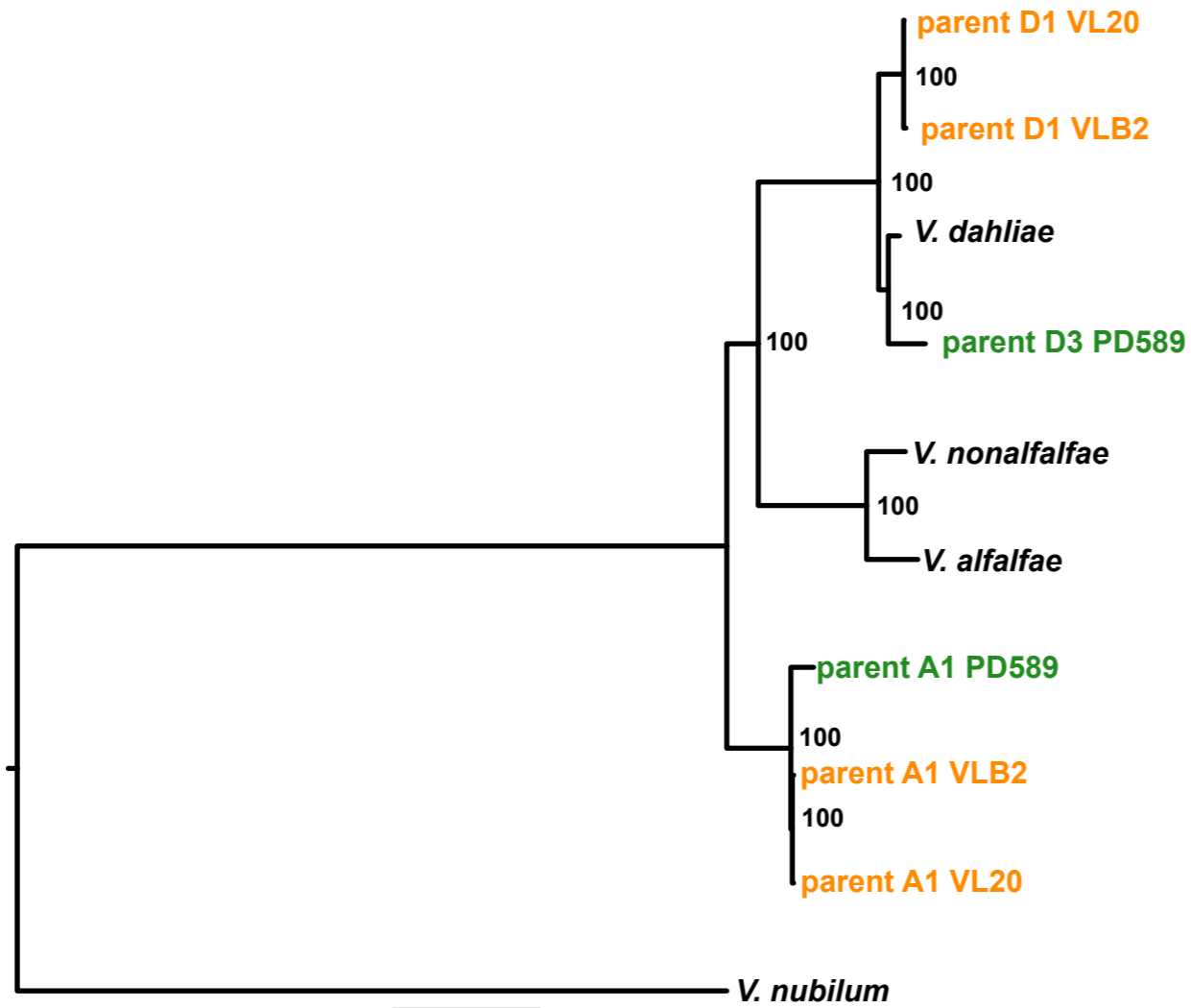

0.02

A1/D1 hybridization

## Mitochondrial genomes

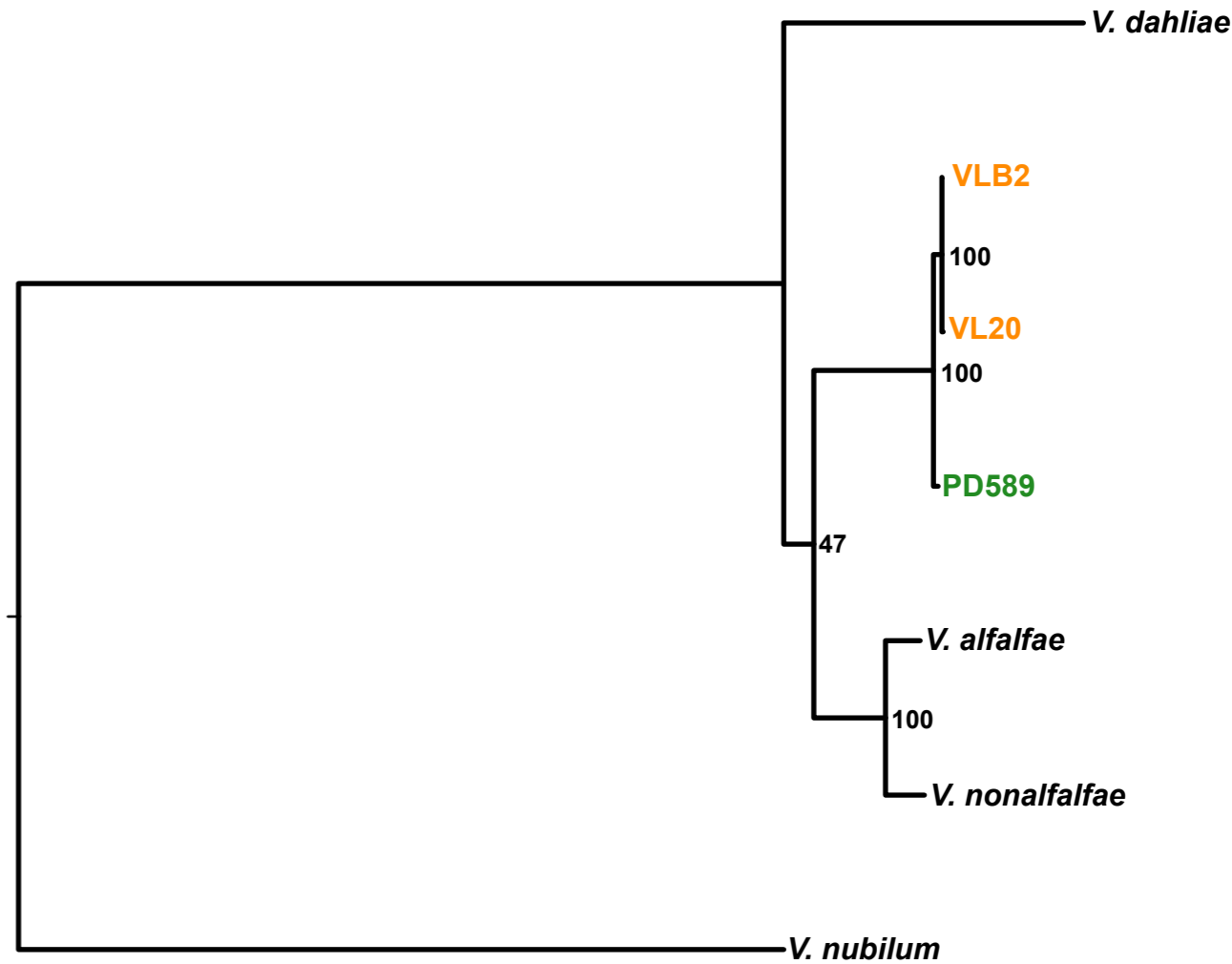

0.006

A1/D3 hybridization

Supplement: FIG S5 [file mbio.01496-21-sf005.pdf]

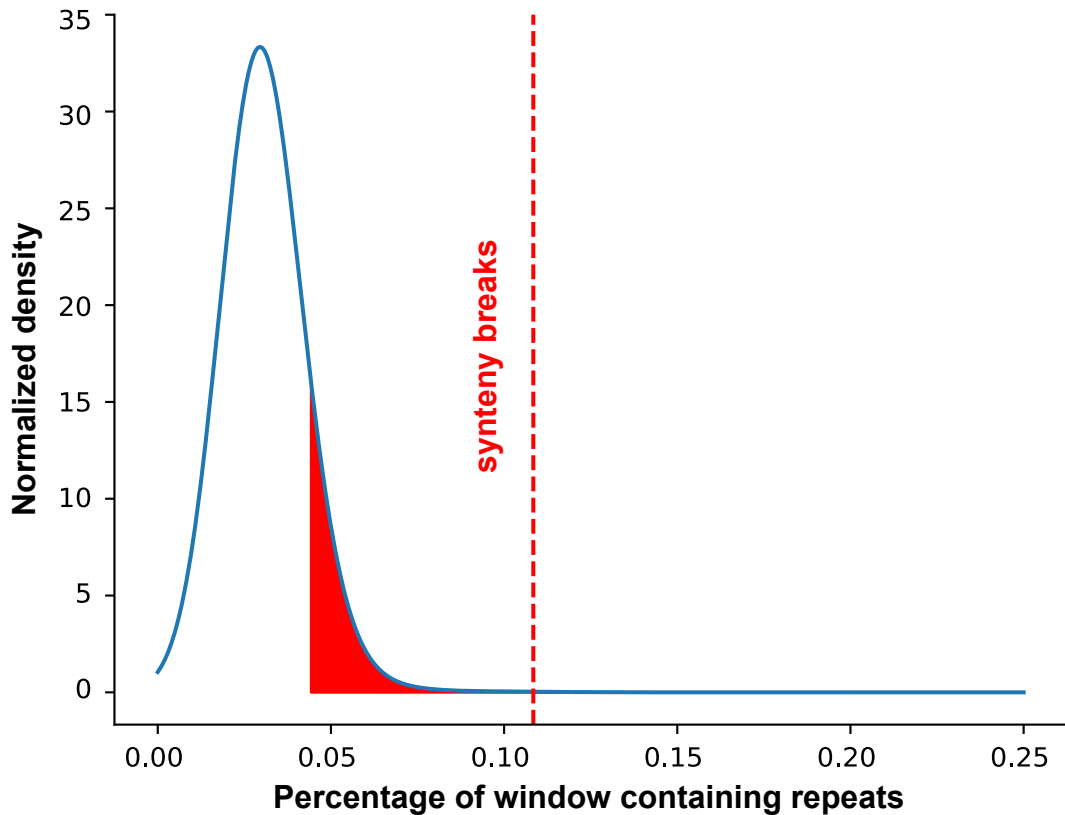

Supplement: FIG S6 [file mbio.01496-21-sf006.pdf]
