# Supplementary material for: The Interspecific Fungal Hybrid Verticillium longisporum Displays Subgenome-Specific Gene Expression
Source: mBio. 2021 Jul 20;12(4):e01496-21. doi: 10.1128/mBio.01496-21 (PMC8406199; doi:10.1128/mBio.01496-21)
Supplement: FIG S2 [file mbio.01496-21-sf002.pdf]

PD589

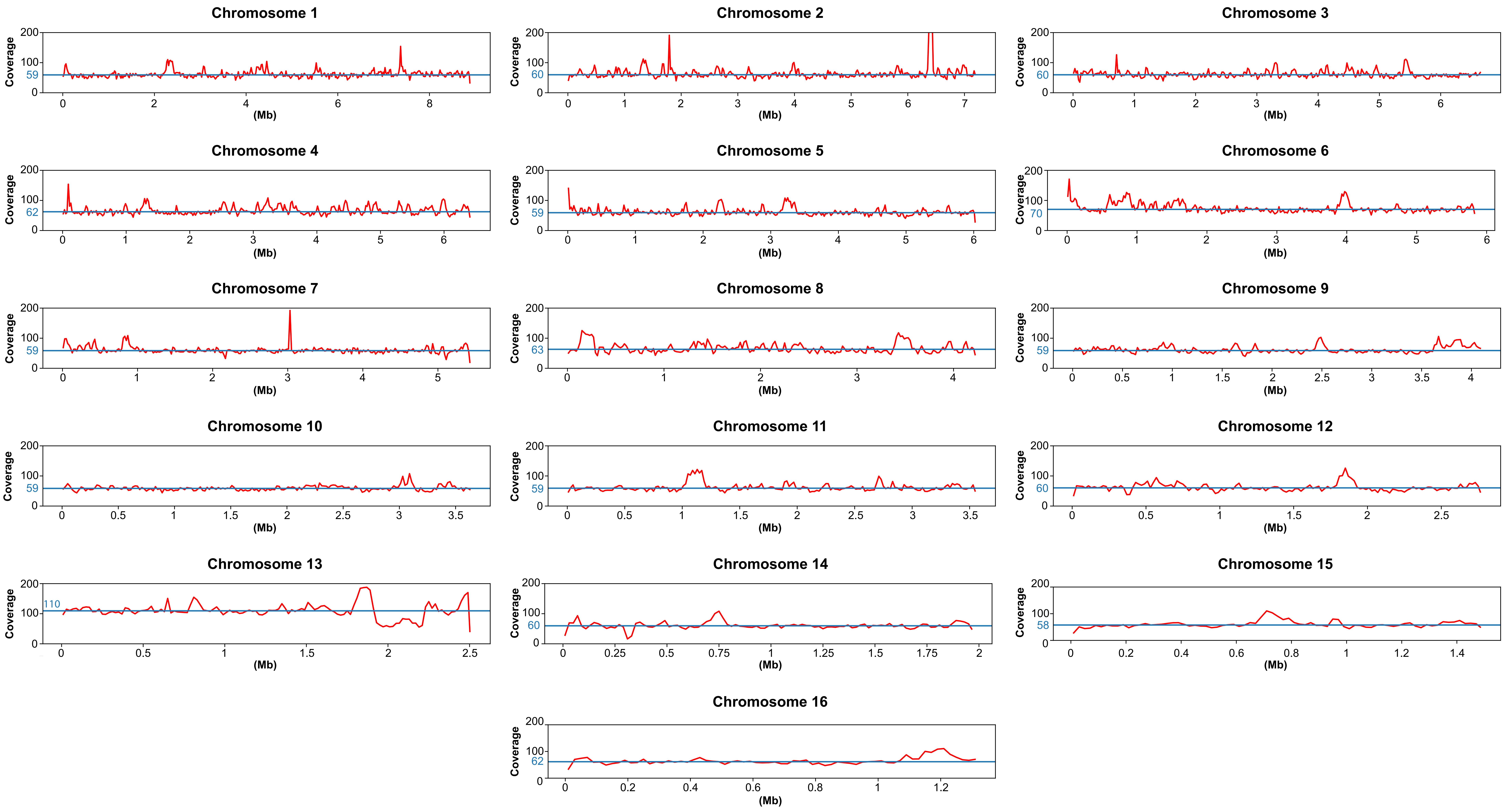

Chromosome 1
Coverage
200
100
59
0
0 2 4 6 8
(Mb)
Chromosome 2
Coverage
200
100
60
0
0 1 2 3 4 5 6 7
(Mb)
Chromosome 3
Coverage
200
100
60
0
0 1 2 3 4 5 6
(Mb)
Chromosome 4
Coverage
200
100
62
0
0 1 2 3 4 5 6
(Mb)
Chromosome 5
Coverage
200
100
59
0
0 1 2 3 4 5 6
(Mb)
Chromosome 6
Coverage
200
100
70
0
0 1 2 3 4 5 6
(Mb)
Chromosome 7
Coverage
200
100
59
0
0 1 2 3 4 5
(Mb)
Chromosome 8
Coverage
200
100
63
0
0 1 2 3 4
(Mb)
Chromosome 9
Coverage
200
100
59
0
0 0.5 1 1.5 2 2.5 3 3.5 4
(Mb)
Chromosome 10
Coverage
200
100
59
0
0 0.5 1 1.5 2 2.5 3 3.5
(Mb)
Chromosome 11
Coverage
200
100
59
0
0 0.5 1 1.5 2 2.5 3 3.5
(Mb)
Chromosome 12
Coverage
200
100
60
0
0 0.5 1 1.5 2 2.5
(Mb)
Chromosome 13
Coverage
200
110
100
0
0 0.5 1 1.5 2 2.5
(Mb)
Chromosome 14
Coverage
200
100
60
0
0 0.25 0.5 0.75 1 1.25 1.5 1.75 2
(Mb)
Chromosome 15
Coverage
200
100
58
0
0 0.2 0.4 0.6 0.8 1 1.2 1.4
(Mb)
Chromosome 16
Coverage
200
100
62
0
0 0.2 0.4 0.6 0.8 1 1.2
(Mb)
