# Supplementary material for: The Interspecific Fungal Hybrid Verticillium longisporum Displays Subgenome-Specific Gene Expression
Source: mBio. 2021 Jul 20;12(4):e01496-21. doi: 10.1128/mBio.01496-21 (PMC8406199; doi:10.1128/mBio.01496-21)
Supplement: TABLE S1 [file mbio.01496-21-st001.doc]

**Table S1. Fractions of individual *Verticillium longisporum* chromosomes that belong to the A1 and D parent.**

| **Chr.** | **VLB2** | | | **VL20** | | | **PD589** | | |
| --- | --- | --- | --- | --- | --- | --- | --- | --- | --- |
|  | **A1** | **D1** | **UND** | **A1** | **D1** | **UND** | **A1** | **D3** | **UND** |
| **1** | 54% | 45% | 1% | 55% | 44% | 1% | 8% | 92% | 0% |
| **2** | 39% | 60% | 1% | 100% | 0% | 0% | 75% | 23% | 2% |
| **3** | 99% | 0% | 1% | 35% | 64% | 0% | 85% | 15% | 1% |
| **4** | 39% | 60% | 1% | 67% | 31% | 2% | 54% | 43% | 3% |
| **5** | 0% | 99% | 0% | 53% | 47% | 0% | 85% | 14% | 1% |
| **6** | 56% | 43% | 1% | 0% | 99% | 1% | 46% | 50% | 4% |
| **7** | 28% | 71% | 0% | 72% | 25% | 2% | 17% | 81% | 1% |
| **8** | 76% | 22% | 2% | 69% | 30% | 1% | 68% | 31% | 1% |
| **9** | 39% | 61% | 0% | 20% | 80% | 0% | 27% | 72% | 1% |
| **10** | 0% | 100% | 0% | 30% | 70% | 1% | 0% | 99% | 1% |
| **11** | 56% | 42% | 2% | 13% | 86% | 1% | 65% | 29% | 6% |
| **12** | 13% | 86% | 1% | 47% | 52% | 1% | 79% | 19% | 2% |
| **13** | 98% | 2% | 1% | 23% | 77% | 1% | 41% | 59% | 0% |
| **14** | 87% | 13% | 1% | 55% | 44% | 2% | 81% | 18% | 2% |
| **15** | 79% | 20% | 1% | 91% | 8% | 1% | 0% | 100% | 0% |
| **16** |  |  |  |  |  |  | 72% | 23% | 5% |
