# Supplementary material for: The Interspecific Fungal Hybrid Verticillium longisporum Displays Subgenome-Specific Gene Expression
Source: mBio. 2021 Jul 20;12(4):e01496-21. doi: 10.1128/mBio.01496-21 (PMC8406199; doi:10.1128/mBio.01496-21)
Supplement: TABLE S2 [file mbio.01496-21-st002.doc]

**Table S2. Expression pattern correlation of genes between *Verticillium dahliae* and *Verticillium longisporum* sub-genomes grown in culture medium.**

|  | **JR2** | **CQ2** | **VLB2 A1** | **VLB2 D1** | **VL20 A1** | **VL20 D1** | **PD589 A1** | **PD589 D3** |
| --- | --- | --- | --- | --- | --- | --- | --- | --- |
| **JR2** | 1.00 | 0.89 | 0.87 | 0.89 | 0.87 | 0.89 | 0.82 | 0.85 |
| **CQ2** | 0.89 | 1.00 | 0.80 | 0.82 | 0.82 | 0.85 | 0.84 | 0.89 |
| **VLB2 A1** | 0.87 | 0.80 | 1.00 | 0.96 | 0.97 | 0.94 | 0.82 | 0.80 |
| **VLB2 D1** | 0.89 | 0.82 | 0.96 | 1.00 | 0.94 | 0.97 | 0.80 | 0.81 |
| **VL20 A1** | 0.87 | 0.82 | 0.97 | 0.94 | 1.00 | 0.96 | 0.84 | 0.81 |
| **VL20 D1** | 0.89 | 0.85 | 0.94 | 0.97 | 0.96 | 1.00 | 0.81 | 0.83 |
| **PD589 A1** | 0.82 | 0.84 | 0.82 | 0.80 | 0.84 | 0.81 | 1.00 | 0.93 |
| **PD589 D3** | 0.85 | 0.89 | 0.80 | 0.81 | 0.81 | 0.83 | 0.93 | 1.00 |

A1 = *V. longisporum* A1 sub-genome, D1 = *V. longisporum* D1 sub-genome and D3 = *V. longisporum* D3 sub-genome. Correlations are calculated with the Spearman’s rank correlation coefficient based on the transcripts per million (tpm) values.
