# Supplementary material for: The Interspecific Fungal Hybrid Verticillium longisporum Displays Subgenome-Specific Gene Expression
Source: mBio. 2021 Jul 20;12(4):e01496-21. doi: 10.1128/mBio.01496-21 (PMC8406199; doi:10.1128/mBio.01496-21)
Supplement: TABLE S3 [file mbio.01496-21-st003.doc]

**Table S3. Functional enrichment analysis of *Verticillium longisporum* genes with differential homeolog expression.**

|  |  | **CULTURE MEDIUM** | | | | | | | | | | | **OILSEED RAPE** | | | | | | |
| --- | --- | --- | --- | --- | --- | --- | --- | --- | --- | --- | --- | --- | --- | --- | --- | --- | --- | --- | --- |
|  |  | **VLB2** | | | | **VL20** | | | | **PD589** | | | **VLB2** | | | | **PD589** | | |
| **GO_term** | **Description** | ***p*-value** | **diff expr** | **all** | ***p*-value** | | **diff expr** | **all** | ***p*-value** | | **diff expr** | **all** | ***p*-value** | **diff expr** | **all** | ***p*-value** | | **diff expr** | **all** |
| 0004497 | monooxygenase activity | 1.23E-03 | 9 | 18 | 2.05E-02 | | 7 | 17 |  | |  |  |  |  |  |  | |  |  |
| 0005506 | iron ion binding | 3.48E-03 | 19 | 70 | 3.71E-04 | | 21 | 70 |  | |  |  |  |  |  | 3.34E-02 | | 6 | 51 |
| 0005975 | carbohydrate metabolic process |  |  |  | 2.05E-02 | | 33 | 174 |  | |  |  |  |  |  |  | |  |  |
| 0006725 | cellular aromatic compound metabolic process |  |  |  | 2.32E-02 | | 4 | 6 |  | |  |  |  |  |  |  | |  |  |
| 0008061 | chitin binding |  |  |  | 4.66E-02 | | 5 | 11 |  | |  |  |  |  |  |  | |  |  |
| 0008237 | metallopeptidase activity | 1.34E-02 | 8 | 20 | 3.58E-02 | | 7 | 19 |  | |  |  |  |  |  |  | |  |  |
| 0016020 | membrane |  |  |  |  | |  |  | 4.39E-02 | | 42 | 198 |  |  |  |  | |  |  |
| 0016491 | oxidoreductase activity | 3.38E-05 | 67 | 329 | 1.33E-04 | | 64 | 325 | 3.55E-03 | | 59 | 270 |  |  |  |  | |  |  |
| 0016614 | oxidoreductase activity, acting on CH-OH group of donors | 3.61E-02 | 6 | 14 | 6.47E-03 | | 8 | 18 |  | |  |  |  |  |  |  | |  |  |
| 0016705 | oxidoreductase activity, acting on paired donors, with incorporation or reduction of molecular oxygen | 1.64E-04 | 19 | 56 | 8.56E-05 | | 20 | 57 |  | |  |  |  |  |  | 3.51E-02 | | 5 | 37 |
| 0020037 | heme binding | 9.11E-05 | 23 | 73 | 1.61E-03 | | 20 | 72 |  | |  |  |  |  |  | 1.25E-02 | | 7 | 56 |
| 0022857 | transmembrane transporter activity | 5.97E-09 | 64 | 246 | 2.59E-06 | | 56 | 238 | 3.07E-03 | | 47 | 193 |  |  |  |  | |  |  |
| 0050660 | flavin adenine dinucleotide binding | 1.70E-03 | 21 | 77 | 3.71E-04 | | 23 | 81 |  | |  |  |  |  |  | 4.91E-04 | | 9 | 58 |
| 0055085 | transmembrane transport | 3.38E-05 | 70 | 347 | 2.73E-04 | | 64 | 334 | 3.07E-03 | | 60 | 271 |  |  |  |  | |  |  |
| 0055114 | oxidation-reduction process | 5.97E-09 | 101 | 474 | 2.59E-06 | | 92 | 471 | 3.80E-02 | | 72 | 378 |  |  |  | 3.60E-04 | | 25 | 362 |
| 0071949 | FAD binding | 1.02E-02 | 13 | 43 | 9.16E-03 | | 13 | 43 | 4.39E-02 | | 11 | 31 |  |  |  | 2.79E-04 | | 7 | 26 |
| **COG** | **Description** | ***p*-value** | **diff expr** | **all** | ***p*-value** | | **diff expr** | **all** | ***p*-value** | | **diff expr** | **all** | ***p*-value** | **diff expr** | **all** | ***p*-value** | | **diff expr** | **all** |
| G | Carbohydrate transport and metabolism | 8.39E-05 | 90 | 509 | 5.02E-06 | | 95 | 511 | 2.58E-02 | | 75 | 421 |  |  |  |  | |  |  |
| Q | Secondary metabolites biosynthesis, transport, and catabolism | 1.95E-10 | 73 | 287 | 1.21E-05 | | 59 | 282 | 1.49E-02 | | 49 | 240 |  |  |  |  | |  |  |
| S | Function unknown |  |  |  |  | |  |  | 1.49E-02 | | 249 | 1576 |  |  |  |  | |  |  |
| V | Defence mechanisms |  |  |  |  | |  |  | 1.79E-02 | | 10 | 28 |  |  |  |  | |  |  |

Gene Ontology (GO) terms and Clusters of Orthologous Groups (COGs) that are significantly enriched in genes with differential homeolog expression are displayed. *P*-values were calculated with the Fisher’s exact test and were multiple-testing corrected with the Benjamini-Hochberg method.
