# Supplementary material for: The Interspecific Fungal Hybrid Verticillium longisporum Displays Subgenome-Specific Gene Expression
Source: mBio. 2021 Jul 20;12(4):e01496-21. doi: 10.1128/mBio.01496-21 (PMC8406199; doi:10.1128/mBio.01496-21)
Supplement: FIG S7 [file mbio.01496-21-sf007.pdf]

Real-time PCR validation of RNAseq data

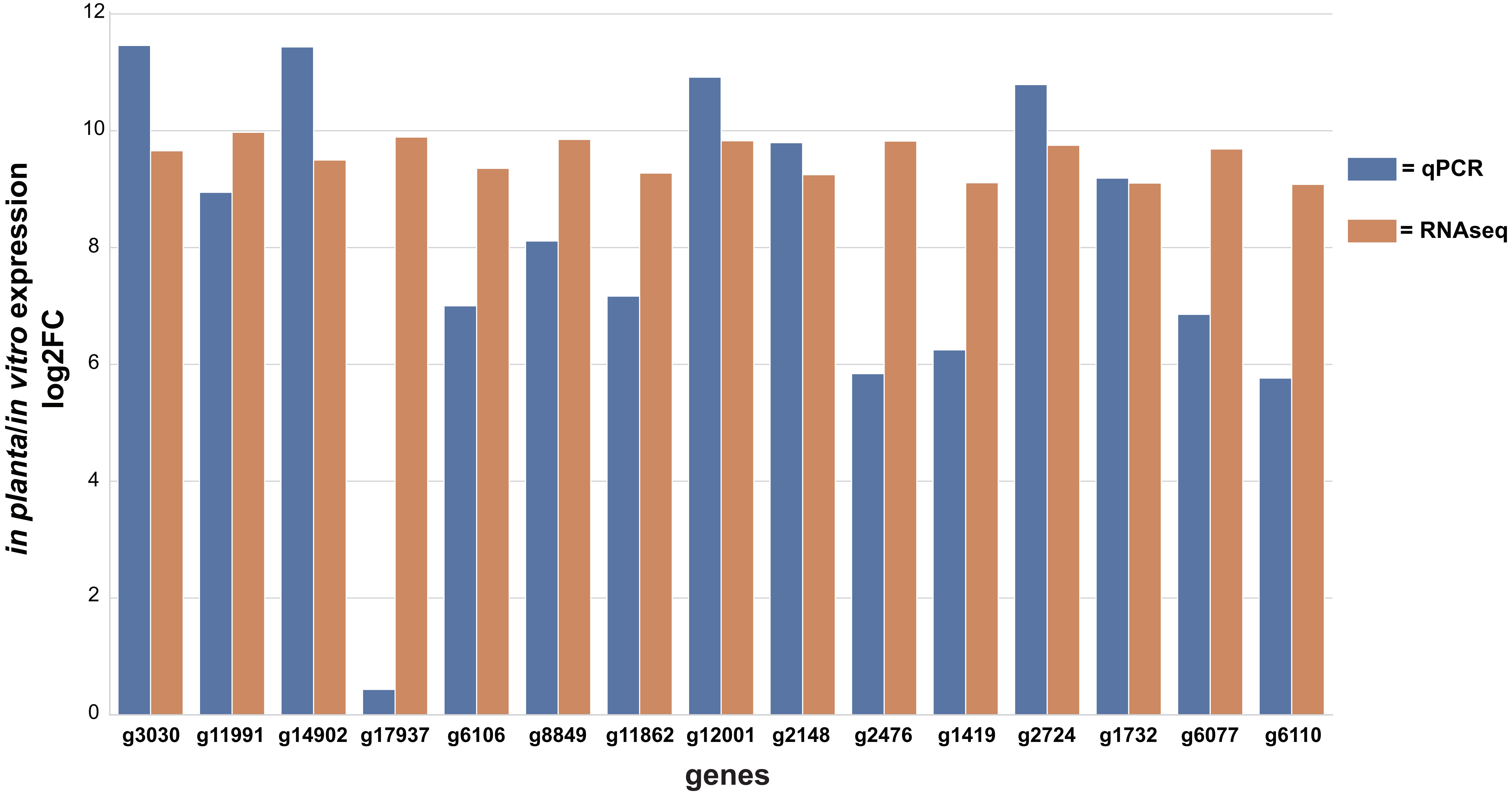

Gene	Forward Primer Sequence (5'→3')	Reverse Primer Sequence (5'→3')	Pfam 1		Pfam2	
PD589_g3030	GCGCAACATCAGCTACTTCA	TTGATCCGCCTCTCCATACC				
PD589_g11991	ACTATGATCTTCGCCGCTCA	AGTCTCTTCTTCTGGCCACC	PF01061	ABC-2 type transporter	PF00005	ABC transporter
PD589_g14902	GGCTCGCACAAGAAAACAGA	GCATTGCGATCGAGGAAGTT	PF07690	Major Facilitator Superfamily		
PD589_g17937	TGGATTGGAACCGACGTGTA	CTGCGAAGGTGAAAGTCTGG				
PD589_g6106	CGATCGATGACATCCGCTTC	CGGTAAGTGCGGTCAATCTG	PF07470	Glycosyl Hydrolase Family 88		
PD589_g8849	ACTACAACCCTTACCGCCTC	TTGTCACGGAAGCGAGAGAT	PF00840	Glycosyl hydrolase family 7		
PD589_g11862	TACTGATCGGGCATTCGTCA	TCCAAGCTCGTCATCAAGGT	PF02734	DAK2 domain	PF02733	Dak1 domain
PD589_g12001	CGATTGGACATGGACACGAC	AGAGAGCAGCGAGTCAAAGT	PF08030	Ferric reductase NAD binding domain	PF08022	FAD-binding domain
PD589_g2148	GCACGGGATCCATTCTGAAC	CGTTCTTGGAGCTGACGATG	PF00295	Glycosyl hydrolases family 28	PF00295	Glycosyl hydrolases family 28
PD589_g2476	GTACGACCGACAATGGCTTC	ACCGTCAATGTCCTCGTTCT	PF00067	Cytochrome P450		
PD589_g1419	CAACCGCAACTGGAACTTCA	GCCAGTCGATGAAGAGCTTG	PF00246	Zinc carboxypeptidase		
PD589_g2724	CAAACCCCTCTTTTGCGGAA	AGAAAGCAACCCGGTATCCA	PF06609	Fungal trichothecene efflux pump (TRI12)		
PD589_g1732	AAGAGGAAGTCGGACGGC	GTGATATCAGACGGGTTGCC				
PD589_g6077	ATCGGTGTTCCCATGTTCCT	AACGTTCCGAGAAGACCCAT				
PD589_g6110	AGCAACCTTATCAACGGGGA	TAACGATGCGACTCCTCTCC	PF00171	Aldehyde dehydrogenase family		
VdGAPDH	CGAGTCCACTGGTGTCTTCA	CCCTCAACGATGGTGAACTT				
